# Synthesis of chimera oligopeptide including furanoid β-sugar amino acid derivatives with free OHs: mild but successful removal of the 1,2-*O*-isopropylidene from the building block

**DOI:** 10.1007/s00726-020-02923-3

**Published:** 2021-02-09

**Authors:** Kim Hoang Yen Duong, Viktória Goldschmidt Gőz, István Pintér, András Perczel

**Affiliations:** 1 Laboratory of Structural Chemistry and Biology, Institute of Chemistry, ELTE Eötvös Loránd University, Pázmány P. stny. 1/A, Budapest, 1117 Hungary; 2 MTA-ELTE Protein Modeling Research Group, ELTE Eötvös Loránd University, Pázmány P. stny. 1/A, Budapest, 1117 Hungary

**Keywords:** Sugar amino acids, β-peptides, Foldamers, Ribofuranuronic acids, 1,2-*O*-Isopropylidene removal, Chimera peptides

## Abstract

Complementary to hydrophobic five membered ring β-amino acids (e.g. ACPC), β-sugar amino acids (β-SAAs) have found increasing application as hydrophilic building blocks of foldamers and α/β chimeric peptides. Fmoc-protected β-SAAs [e.g. Fmoc-RibAFU(ip)-OH] are indeed useful Lego elements, ready to use for SPPS. The removal of 1,2-OH isopropylidene protecting group increasing the hydrophilicity of such SAA is presented here. We first used N_3_-RibAFU(ip)-OH model compound to optimize mild deprotection conditions. The formation of the 1,2-OH free product N_3_-RibAFU-**OH** and its methyl glycoside methyl ester, N_3_-RibAFU(Me)-**OMe** were monitored by RP-HPLC and found that either 50% TFA or 8 eqv. Amberlite IR-120 H^+^ resin in MeOH are optimal reagents for the effective deprotection. These conditions were then successfully applied for the synthesis of chimeric oligopeptide: -GG-**X**-GG- [X=RibAFU(ip)]. We found the established conditions to be effective and—at the same time—sufficiently mild to remove 1,2-*O*-isopropylidene protection and thus, it is proposed to be used in the synthesis of oligo- and polypeptides of complex sequence combination.

**Electronic supplementary material:**

The online version of this article (10.1007/s00726-020-02923-3) contains supplementary material, which is available to authorized users.

## Introduction

**Fig. 1:**
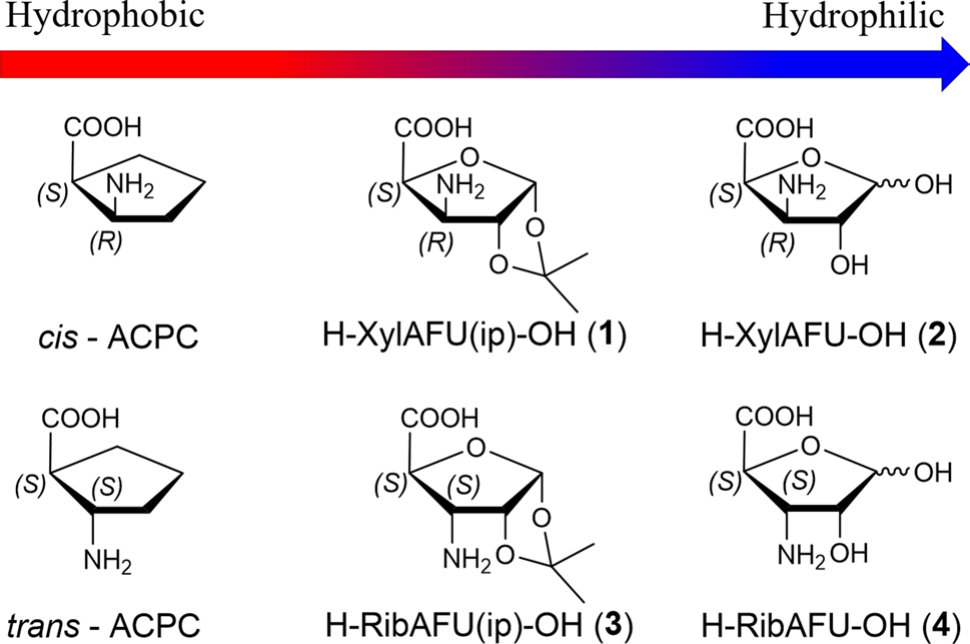
Some hydrophobic β-AAs, the *cis*- and the *trans*-ACPC and their hydrophilic furanoid analogues (β-SAAs): H-XylAFU(ip)-OH (**1**), H-XylAFU-OH (**2**), H-RibAFU(ip)-OH (**3**) and H-RibAFU-OH (**4**)

Oligopeptides containing β-amino acids (β-peptides) have favorable backbone folding properties as foldamers (Gellman 1998; Hill et al. 2001; Seebach et al. 1996). Numerous oligomers made from diastereomers of 2-aminocyclopentanecarboxylic acid (ACPC) (Abraham et al. 2010; Appella et al. 1999a; Martinek et al. 2002) and 2-aminocyclohexanecarboxylic acid (ACHC) (Appella et al. 1996; Appella et al. 1999b; Hetényi et al. 2005) are regarded as benchmark nanosystems incorporating cyclic β-amino acids. However, the hydrophobic character enhanced by ACPC and ACHC residues is a serious drawback during their potential physiological application. In fact, their homooligomers are insoluble in water (Hetényi et al. 2009). This can be amended by introducing sugar amino acids (SAAs) (Risseeuw et al. 2009, 2013) which are more hydrophilic by nature. It has been shown particularly, both for five- and six-membered cyclic SAAs (H-SAA-OHs) to behave as appropriate building blocks (Nagy et al. 2017; Csordás et al. 2016; Goldschmidt Gőz et al. 2018; Suhara et al. 2006; Chandrasekhar et al. 2004; Gruner et al. 2002a, b; Pandey et al. 2011). Furanoid β-SAAs, e.g., 3-amino-3-deoxy-d-furanuronic acids (AFUs), primarily, d-*xylo* (**1**, **2**) and d-*ribo* (**3**, **4**) epimeric pairs (Nagy et al. 2017) are hydrophilic analogs of *cis*- and *trans*-ACPC (Fig. 1). The cost-effective synthesis of oligopeptides incorporating both furanoid and pyranoid β-SAAs were recently done in our group (Csordás et al. 2016; Nagy et al. 2019).

The stable protection of the hydroxyl groups of β-SAA is essential during the synthesis of oligo- and polypeptides. Afterwards, the removal of their protecting groups is of a special interest to enhance the hydrophilicity of the parent chimera peptides (Suhara et al. 2006; Schweizer 2002; Gruner et al. 2002a, b; Chakraborty et al. 2002). In principle, the hydrophilic moieties ensure the better compatibility with the living organism of aqueous media. In addition, the introduction of more hydrophilic residues makes these types of β-SAA building blocks tunable and more versatile and thus, enhanced biocompatibility could be achieved, otherwise crucial for drug delivery and biomedical applications.

**Scheme 1:**
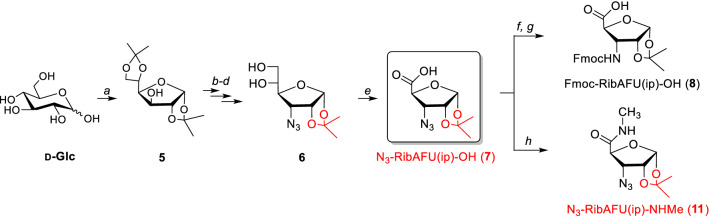
The synthesis of the furanoid β-SAA, Fmoc-RibAFU(ip)-OH (**8**) starts from d-Glc, turned subsequently into the azido derivative (**7**). Reagents and conditions: *a* (CH_3_)_2_CO, H_2_SO_4_; *b* NaH, Im_2_SO_2_, DMF; *c* NaN_3_, Bu_4_NBr, toluene; *d* 75% AcOH; *e* 1. NaIO_4_, MeOH-H_2_O, 2. KMnO_4_, 50% AcOH; *f* 10% Pd/C, MeOH (H-Cube^®^); *g* Fmoc-OSu, THF, MeOH-H_2_O; *h* CH_3_NH_2_.HCl, EDC.HCl, DIEA, HOBt, DCM

Both the N_3_-RibAFU(ip)-OH (**7**) and Fmoc-RibAFU(ip)-OH (**8**) β-SAA have their 1,2-*O*-isopropylidene protecting groups originating from 1,2;5,6-di-*O*-isopropylidene-d-glucofuranose (**5**) (Scheme 1) (Nagy et al. 2017). Note that, the original 5,6-*O*-isopropylidene group was removed in one of the intermediary steps. The selective deprotection of 5,6-*O*-isopropylidene acetals of d-Glc is described using protic acids: e.g., H_2_SO_4_ (Rbaa et al. 2020; Miljkovic 2009; Sukumar et al. 1986), HClO_4_ on silica gel (Agarwal and Vankar 2005), polyphosphoric acid on silica gel (Nikam and Gore 2020) or AcOH (Ma et al. 2020; Pikas et al. 2020; Ferreira et al. 2020; Ravn et al. 2019; Gruner et al. 2002a, b; Yadav, Chander and Reddy 1992). The application of some Lewis acids was also reported, such as FeCl_3_·6H_2_O/SiO_2_ (Kim et al. 1986), CuCl_2_·2H_2_O (Iwata and Ohrui 1981), Zn(NO_3_)_2_·6H_2_O (Vijayasaradhi, Singh and Aidhen 2000), Yb(OTf)_3_·H_2_O (Yadav et al. 2001) just as BiCl_3_ (Swamy and Venkateswarlu 2002). Alternative methods for the selective removal of the isopropylidene protection under relatively mild conditions were studied more recently as aqueous *tert*-butyl hydroperoxide (Maddani and Prabhu 2011), CBr_4_-photoirradiation (Chen et al. 2004) and acid zeolites (Rauter et al. 2010). The removal of 2,3-*O*-isopropylidene acetals is also presented in acidic conditions of TFA (Bornaghi et al. 2004; Ganapati and Arvind 2020; Ferreira et al. 2020; Gelin et al. 2020; Pogula et al. 2020; Ahmed-Belkacem et al. 2020), AcOH (Decultot et al. 2020), HCl (Ko et al. 2017) or BCl_3_ (Yamamoto et al. 2019; Yoo et al. 2018). For deprotection of 1,2-*O*-isopropylidene moieties, fewer approaches were mentioned, including H_2_SO_4_ (Masamune et al. 2001; Yanaisaka et al. 1970), HCl (Sorensen et al. 2001; Yanaisaka et al. 1970), TsOH (Yuan et al. 2020; Sukumar et al. 1986; Rosenthal and Cliff 1980), aqueous TFA (Piccini et al. 2020; Fernandez-Bolanos and Lopez 2007) or cationic exchange resins (e.g. Dowex-50 H^+^) (Weber et al. 1986; Fleet and Smith 1985). The deprotection conditions for various di-*O*-isopropylidene of sugar scaffolds are summarized in Table 1.

**Table 1:** Using of di-*O*-isopropylidene deprotection for sugar ring in various scaffolds and systems

Ring size	Isopropylidene protection		
	1,2-*O*-	2,3-*O*-	5,6-*O*-
5-membered	TFA/H_2_O (Fernandez-Bolanos and Lopez 2007) TFA/DCM-H_2_O (Piccini et al. 2020) Dowex 50 W-X8 resin/MeOH (Weber et al. 1986; Fleet and Smith 1985) TsOH/AcOH-Ac_2_O (Rosenthal and Cliff 1980) TsOH/MeCN-H_2_O (Sukumar et al. 1986) TsOH/THF-toluene (Yuan et al. 2020) *cc*. H_2_SO_4_/AcOH-Ac_2_O (Yanaisaka et al. 1970; Masamune et al. 2001) Dry HCl/ether-AcCl (Yanaisaka et al. 1970) HCl/MeOH-H_2_O-DCM (Sorensen et al. 2001) AcCl/MeOH-H_2_O-DCM (Sorensen et al. 2001)	TFA/H_2_O (Gelin et al. 2020; Ganapati and Arvind 2020; Ferreira et al. 2020; Pogula et al. 2020; Ahmed-Belkacem et al. 2020) TFA/H_2_O-DMSO (Bornaghi et al. 2004) TFA/H_2_O-DCM (Ganapati and Arvind 2020) TFA/DCM (Ganapati and Arvind 2020) BCl_3_/DCM (Yamamoto et al. 2019; Yoo et al. 2018) AcOH/H_2_O (Decultot et al. 2020) *cc*. HCl (Ko et al. 2017)	AcOH/H_2_O (Ma et al. 2020; Pikas et al. 2020; Ferreira et al. 2020; Ravn et al. 2019; Gruner et al. 2002a, b; Yadav, Chander and Reddy 1992) H_2_SO_4_/MeOH (Sukumar et al. 1986) H_2_SO_4_/EtOH (Rbaa et al. 2020) PPA·SiO_2_/MeCN (Nikam and Gore 2020) HClO_4_·SiO_2_/MeOH (Agarwal and Vankar 2005) FeCl_3_·6H_2_O/SiO_2_ in CHCl_3_ (Kim et al. 1986) Yb(OTf)_3_·H_2_O/MeCN (Yadav et al. 2001) Zn(NO_3_)_2_·6H_2_O/MeCN (Vijayasaradhi, Singh and Aidhen 2000) BiCl_3_/MeCN-DCM (Swamy and Venkateswarlu 2002) *tert*-butyl hydroperoxide (TBHP)/H_2_O (Maddani and Prabhu 2011) CBr_4_/MeOH (Chen et al. 2004)

Ring size	Isopropylidene protection				
	1,2-*O*-	2,3-*O*-	3,4-*O*-	4,5-*O*-	4,6-*O*-
6-membered	TFA/H_2_O (Ahmed et al. 2020; Ferreira et al. 2020)	TFA/DCM-H_2_O (Piccini et al. 2020) TFA/DCM (Vlahovicek-Kahlina et al. 2018) TFA/H_2_O (Vlahovicek-Kahlina et al. 2018) BCl_3_/DCM (Yamamoto et al. 2019; Yoo et al. 2018) AcOH/H_2_O (Tian et al. 2020; Sun et al. 2020; Xiao et al. 2020; Li et al. 2020)	TFA/H_2_O (Ahmed et al. 2020; Ferreira et al. 2020) DTT/CSA-DCM (Sun et al. 2020)	TFA/DCM (Vlahovicek-Kahlina et al. 2018) TFA/H_2_O (Vlahovicek-Kahlina et al. 2018)	BCl_3_/DCM (Yamamoto et al. 2019; Yoo et al. 2018) TsOH.H_2_O/MeOH-DCM (Shuai et al. 2020)

Although several papers described various conditions for the deprotection of isopropylidene acetals, no case of such 1,2-*O*-isopropylidene removal from SAAs oligopeptides is known. Only 2,3-*O*-isopropylidene removal was reported, when cyclic dimer of protected 5-aminomethyl-3,4-dihydroxy-tetrahydrofuran-2-yl-acetic acid (H-SAA-OH) without 2,3-*O*-isopropylidene acetal was formed in aqueous TFA (Bornaghi et al. 2004). In a patent of antibody–drug conjugates, a linker as MC-SAA-Phe-Cit-APEA containing 5-azidomethyl-3,4-dihydroxy-tetrahydrofuran-2-yl-acetic acid (N_3_-SAA-OH) was synthesized and the 2,3-*O*-isopropylidene protection was removed with aqueous HCl, following the peptide synthesis (Ko et al. 2017). Linkers having high hydrophilic SAAs were designed to reduce significantly or even avoid the use of organic solvents during the conjugation process. This can enhance high conjugation efficiency and the stability of conjugate products, which is important in drug development.

Therefore, it seems essential to develop applicable approaches for peptides having 1,2-*O*-isopropylidene protected SAA building blocks. The main goals we set out to: (1) free the furanoid 1,2-OHs to increase the hydrophilicity of the molecule and (2) form methyl glycoside to prevent furanoid ring opening. Consequently, we synthesized our model oligopeptide Ac-GG-**X**-GG-R (Nagy et al. 2019) this time with X=RibAFU(ip) and R=OH or NH_2_ and the removal conditions of the 1,2-*O*-isopropylidene protecting group were successfully probed.

## Results and discussion

Realizing the two main goals, we took into account the essential criteria in both cases: only the planned reactions might happen and unwished transformations in the polypeptide chain (chain scission, rearrangement, elimination, etc.) cannot occur.

To work out the conditions suitable for removal of 1,2-*O*-isopropylidene protection, the easily accessible intermediate N_3_-RibAFU(ip)-OH (**7**) (Scheme 1) was selected and probed. In addition, N_3_-RibAFU(ip)-**NHMe** (**11**) (Nagy et al. 2017), the amide derivative of **7**, was also used as proper model to provide the possibility of the comparison with the related oligopeptides.

An efficient multigram synthesis of the sugar amino acid Fmoc-RibAFU(ip)-OH (**8**) was completed previously from d-Glc (Nagy et al. 2017). The deprotection of the 5,6-*O*-isopropylidene moiety was achieved with diluted acetic acid (Scheme 1, step *d*). Then, N_3_-RibAFU(ip)-OH (**7**) was obtained from **6** and in the final steps Fmoc-RibAFU(ip)-OH (**8**) product was achieved. We have shown that Fmoc-RibAFU(ip)-OH (**8**) is a suitable monomer for solid-phase peptide synthesis (SPPS), as both the 1,2-OHs and the amino function are protected (Nagy et al. 2019).

**Scheme 2:**
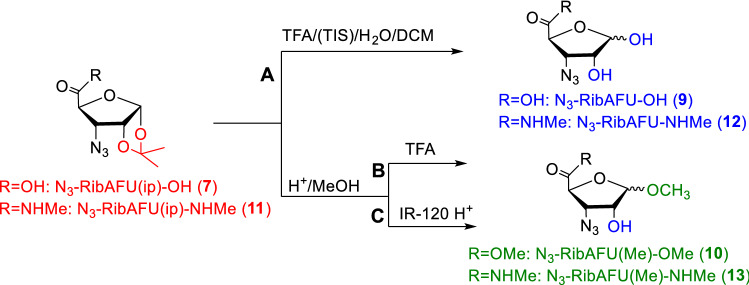
Derivatives **7** and **11** used for optimizing conditions of the 1,2-*O*-isopropylidene removal. Route **A** (see SFig. 22, SFig. 23, SFig. 24, SFig. 26); Route **B** (SFig. 27); Route **C** (Fig. 2, Fig. 3, Table 2, SFig. 20, SFig. 21, SFig. 25, STable 1, STable 2)

The deprotection was carried out under strong acidic conditions (Scheme 2) and the reactions were followed by RP-HPLC. Chromatograms were well resolved and clearly showed the steadily decrease in concentration of the starting **7** (retention time 11.9 min).

In Route **A**: Compound **7** was dissolved in the mixture of TFA in DCM of 50%, 70% and 90% concentrations in parallels together with a small amount of H_2_O (2.5%) and TIS (2.5%) as scavengers. All reactions were carried out at room temperature. Analysis of RP-HPLC diagrams resulted in evaluable data of the reaction with varied concentration of TFA. Since component **9** has hydrophilic character during the deprotection reactions, instead of a conventional C-18 column, Hydro-RP LC column was used for RP-HPLC system. The hydrolysis of **7** (retention time 21.6 min) was almost completed and no intermediate was detected (SFig. 22-24). The new signal at 4.3 min was indicated and the reaction mixture was processed to give white oil, however, attempts to isolate a pure product failed.

Due to the free OH-1 in **9** such mixture of the anomers could be expected. This fact was indicated by ESI–MS exhibiting a peak at *m/z* 188.03029 (SFig. 7) of the unprotected carboxylic acid (**9**). Further studies with HILIC LC–MS (Hydrophilic Interaction Chromatography) corroborated the presence of **9** in the mixture. In these chromatograms, the signal at 2.13 min was assigned to the starting **7** based on the ESI–MS *m/z* 228.06094 [M−H]^−^ (SFig. 14). Furthermore, the signal at 10.46 min was attributed to the product **9** in accordance with ESI–MS *m/z* 188.03057 [M−H]^−^ (SFig. 15). This expected product (**9**) might be also highly unstable, as solely the related *O*-protected derivatives were described in the literature (Nagy et al. 2017; Csordás et al. 2016; Gruner et al. 2002a, b; Masamune et al. 2001; Dauban et al. 1996). Both the starting **7** and the product **9** could be detected with their exact masses, however, the retention time of **7** was short and the peak shape of **9** was not sufficient sharp. Indeed, the separation has to be further optimized to obtain better quality chromatographic peaks, thus, the integration and comparison can be made for following the reactions.

Analogue reaction of N_3_-RibAFU(ip)-**NHMe** (**11**) in TFA/DCM (50%) with TIS (2.5%) and H_2_O (2.5%) was stirred at room temperature for 1 h to be completed according to TLC and RP-HPLC (SFig. 26). The signal of the expected product **12** appeared at about 2 min and indicated the mixture of products also in this case. Working up the reaction mixture gave a solid which could not be separated to give pure anomers. Formation of more products was attributed to the free OH-1 in **12**—as in the case of **9**. Additional reasons for the dispersity of the peak at 2 min was revealed by ESI–MS exhibiting the isotopic peak of an isobaric contaminant at *m/z* 203.822 beside of the peak of the mixture of **12** at *m/z* 203.07803 (SFig. 10).

In Route **B**: Compound **7** was dissolved in the solution of TFA/dried MeOH in parallel concentrations of 30%, 50% and 70%. The mixtures were kept for 18 h at room temperature then were processed to give oily products from each sample. Analysis with RP-HPLC revealed complex mixtures in all cases (SFig. 27). Only the decreasing signal of the starting **7** was unambiguously assigned between 11.7 and 12 min, respectively, depending on the TFA concentration. Further components—intermediates and side products—were detected in all cases. The formation of the intermediate **14** in the reaction mixture of concentration of 30% TFA was identified (SFig. 5). Attempted separation of the components failed from the oily mixtures, thus, Route **B** was not continued.

**Fig. 2:**
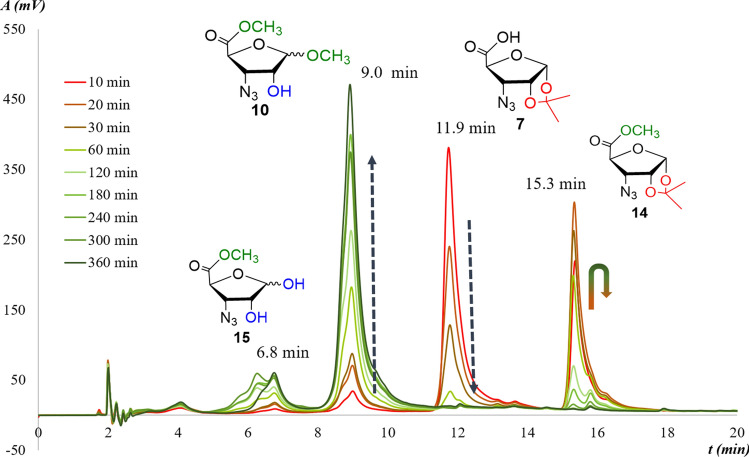
The 1,2-*O*-isopropylidene removal from N_3_-RibAFU(ip)-OH as function of the time resolved by RP-HPLC: compound **7** treated with 8 eqv. of Amberlite IR-120 H^+^ resin in MeOH at 60 °C. Decreasing concentration of the starting **7** and the increasing one of the main product (**10**) were assigned with ^1^H-NMR and MS data

In Route **C**: These reactions of the starting **7** were carried out under heterogeneous conditions: Amberlite IR-120 H^+^ resin was used as the acid component in dried MeOH. The mol ratio was in parallel reactions: 2, 4, 8 and 12 eqv. The mixtures were kept at 60 °C, 40 °C and at room temperature till to the reaction was complete. Analysis of RP-HPLC chromatograms revealed that 1,2-*O*-isopropylidene deprotection on **7** (11.9 min) occurred, parallel, with the formation of the methyl ester methyl furanoside (**10**) as final main product (9.0 min). Besides, two further peaks were detected at 6.8 min and at 15.3 min, respectively. These were reasonably assigned to the temporary intermediate methyl esters (**14** and **15**). The best results were obtained from the reaction with 8 eqv. Amberlite IR-120 H^+^ at 60 °C when almost complete transformation occurred in 360 min (Fig. 2). Parallel reactions at 40 °C and at room temperature terminated after 1 day and 4 days, respectively.

**Fig. 3:**
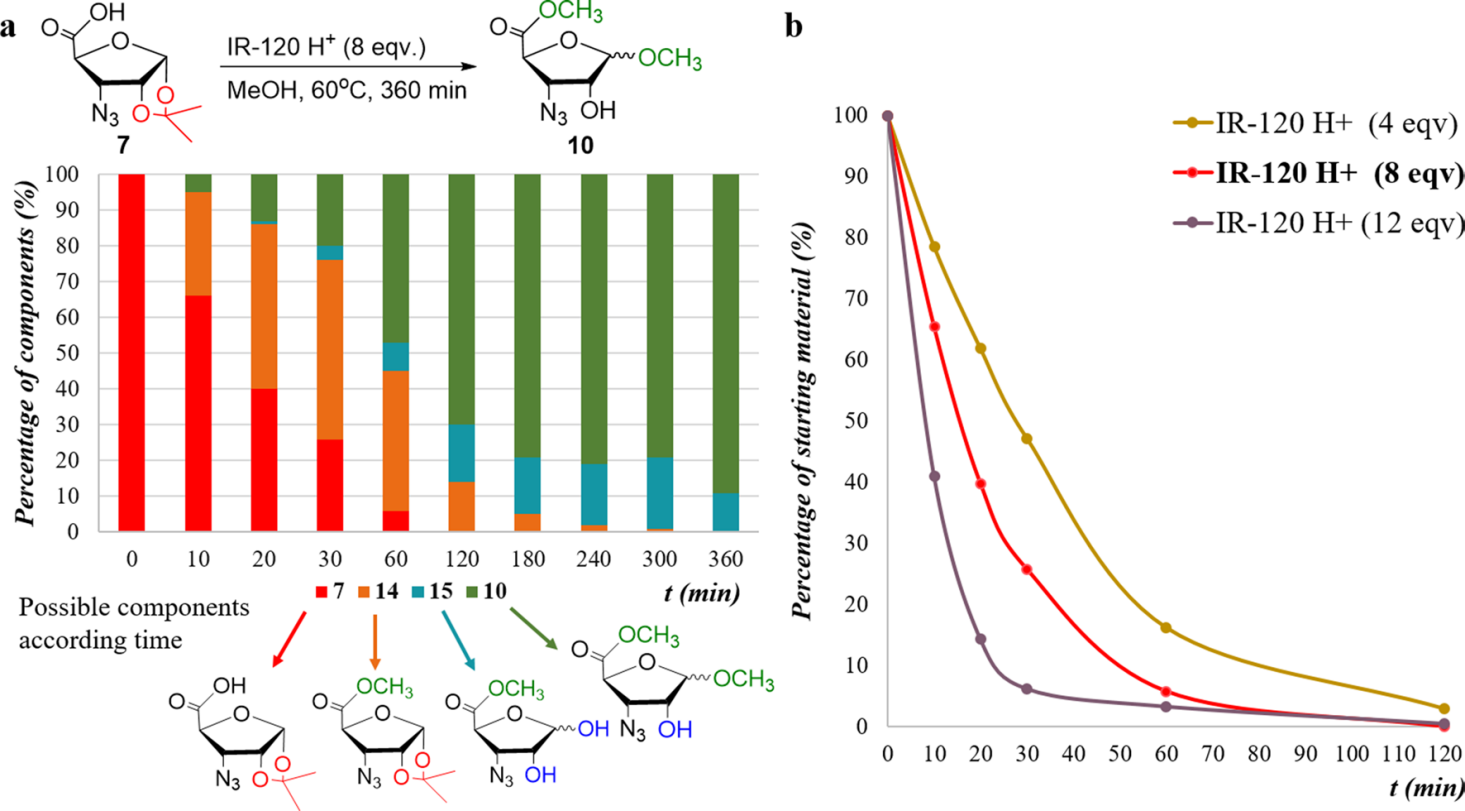
**a** The gradual concentration changes of the starting **7**, intermediates (**14** and **15**) and the final product (**10**) as function of time (0 → *t*(min) → 360) at 60 °C, with 8 eqv. Amberlite IR-120 H^+^ in MeOH. **b** The concentration decrease of the starting **7** as function of time (0 → *t*(min) → 120) at 60 °C, with different equivalents of Amberlite IR-120 H^+^ in MeOH

The conversion to product **10** was near to 90% in each case (Table 2, STable 1, STable 2). Quantitative analysis of the RP-HPLC diagram of the best experiment revealed the disappearance of the starting **7** after 60 min, and that of the intermediate **14** after 240 min (Fig. 3). The mixture of products contained mainly the final product **10** (89%) and also intermediate **15** (~ 10%; not isolated) (Table 2).

**Table 2:** Changes of the concentrations in the starting compound N_3_-RibAFU(ip)-OH (**7**) and in the main product N_3_-RibAFU(Me)-OMe (**10**) at different temperatures with Amberlite IR-120 H^+^ (8 eqv.) in MeOH

Time (min)	Starting azido derivative 7 (%)			Unprotected product 10 (%)		
	60 °C	40 °C	RT	60 °C	40 °C	RT
10	66	88	94	5	0	0
20	40	76	89	13	0	0
30	26	64	83	20	0	0
60	6	37	67	48	6	0
**120**	n.d.^**a**^	13	41	70	19	2
180	n.d.	4	25	79	33	4
240	n.d	2	16	81	43	7
300	n.d	1	10	79	65	10
**360**	n.d	1	7	89	58	13
480	n.d	n.d	3	n.d.	66	21
1440	n.d	n.d	n.d	n.d.	90	57
2880	n.d.	n.d.	n.d.	n.d.	n.d.	79
5760	n.d.	n.d.	n.d.	n.d.	n.d.	88

^a^Compound **7** becomes too low to detect it by RP-HPLC chromatography

**Fig. 4:**
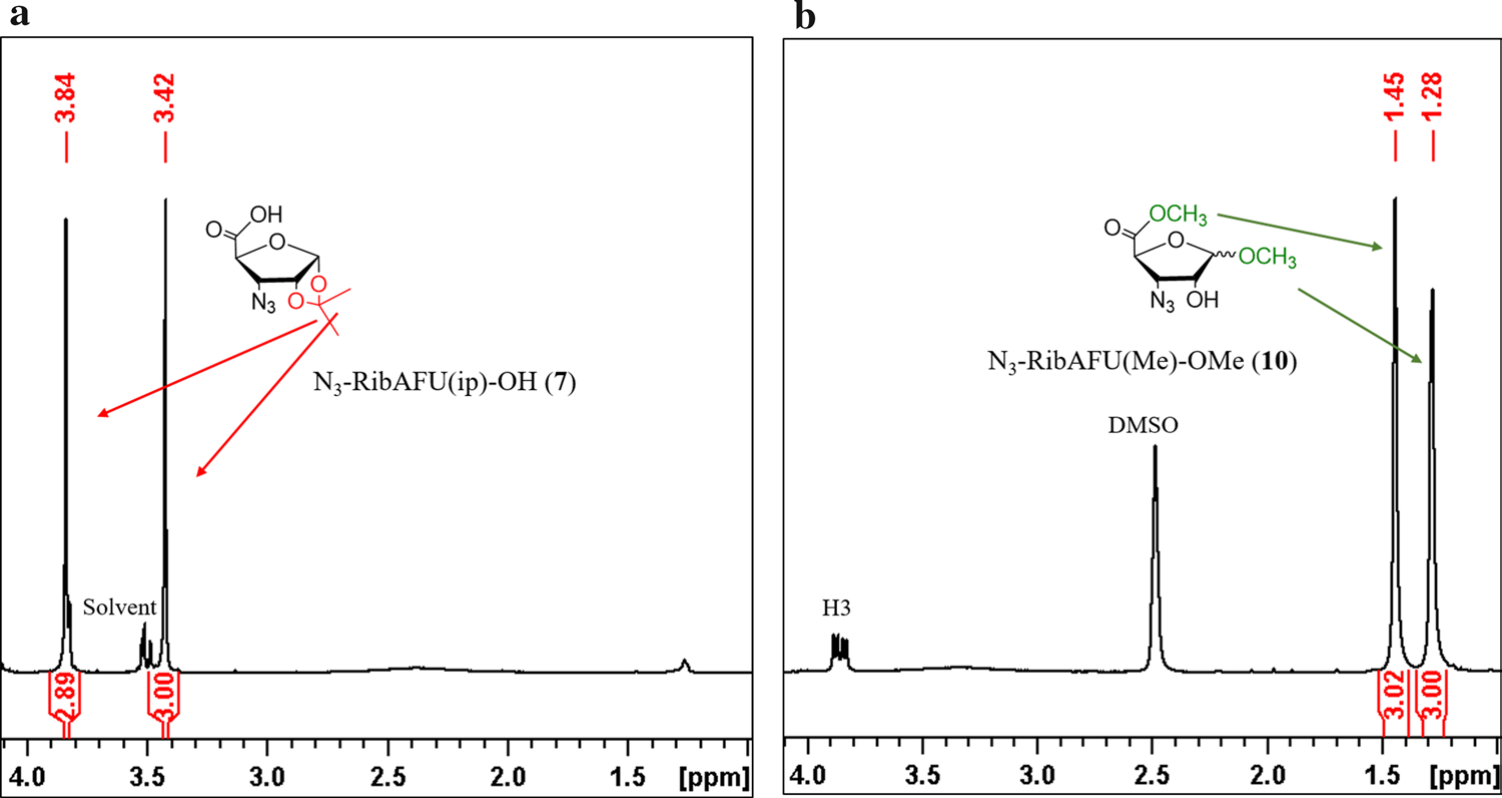
**a** ^1^H NMR spectra of the starting (**7**) showing 1,2-*O*-isopropylidene signals at 1.28 and 1.44 ppm, respectively. **b** The characteristic methyl group resonances of the methyl ester and the methyl *O*-glycoside of (**10**) at 3.42 and 3.84 ppm, respectively

Working up the mixture gave inseparable oil. The ^1^H NMR and ESI–MS were measured with the best mixture. The ^1^H NMR signals of the 1,2-*O*-isopropylidene group of the starting **7** (at *δ* 1.28 and 1.44 ppm) disappeared in the spectrum of the methyl glycoside product (**10**). At the same time, the spectrum of the latter exhibited the characteristic methyl signals of the methyl ester and the methyl *O*-glycoside at 3.84 and 3.42 ppm, respectively (Fig. 4 and SFig. 4). The ratio of the anomers calculated from the H-1 signals shows that α-anomer (^3^*J* coupling of H1-H2: < 1.0 Hz) is the main component and the β-anomer (^3^*J* couplings of H1-H2: 11.5 Hz) is the minor one. ESI–MS exhibited *m/z* [M+Na]^+^ 240.1 for C_7_H_11_N_3_O_5_ supporting the molecular composition of **10**. The small quantity of the minor component did not allow the detailed analysis, however, the evident presence of **15** in the reaction mixture was supported by the mechanism of the complex reaction (Scheme 1).

Analogue reaction of N_3_-RibAFU(ip)-**NHMe** (**11**) was carried out under the best conditions as in the case of **7**: with Amberlite IR-120 H^+^ (8 eqv.)/dried MeOH at 60 °C and completed within 300 min. Analysis of RP-HPLC diagrams revealed that 1,2-*O*-isopropylidene deprotection on **11** (9.9 min) occurred, parallel, with the formation of the expected methyl furanoside (**13**) as final main product (at 4.5 min, SFig. 25). Besides, a second product was observed at 3 min, probably, the 1,2-(OH)_2_ derivative (**12**). Working up the reaction mixture gave an inseparable oily product. The main component of the mixture was identified by ESI–MS which exhibited two strong peaks (*m/z* 217.09303 [M+H]^+^ and *m/z* 239.07487 [M+Na]^+^) corresponding to the structure of **13**. Other two peaks were also measured (*m/z* 203.07725 [M′+H]^+^ and *m/z* 225.19593 [M′+ Na]^+^) which were attributed to the side product **12** in the mixture (SFig. 11).

### Synthesis and deprotection of a model chimera peptide

**Scheme 3:**
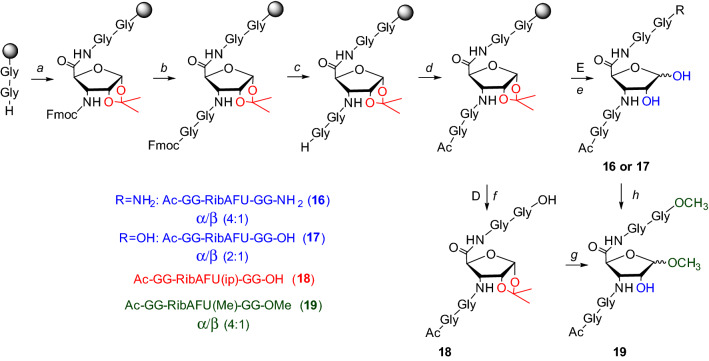
Solid-phase chimera oligopeptide synthesis of -GGXGG- on RAM-Tentagel^®^ or 2-Cl-Trt-Cl resin. The removal of the 1,2-*O*-isopropylidene protection from Ac-GG-RibAFU(ip)-GG- was successful using the following reagents and conditions: *a* Fmoc-RibAFU(ip)-OH (**8**) with PyBOP/DIEA; *b* Fmoc-GG-OH with PyBOP/DIEA; *c* Piperidine (2%)/DBU (2%) in DMF; *d* Ac_2_O:DIEA:DMF (1:1.2:3); *e* TFA (50%)/DCM (45%)/TIS (2.5%)/H_2_O (2.5%); *f* AcOH:MeOH:DCM (1:1:8); *g* IR-120 H^+^ (8 eqv.)/MeOH, 60 °C, 6 h (see SFig. 19); *h* IR-120 H^+^ (8 eqv.)/MeOH, 60 °C, 3 h from compound **17** (see SFig. 18). Ratios of α/β anomers were determined by HILIC LC–UV–MS

**Fig. 5:**
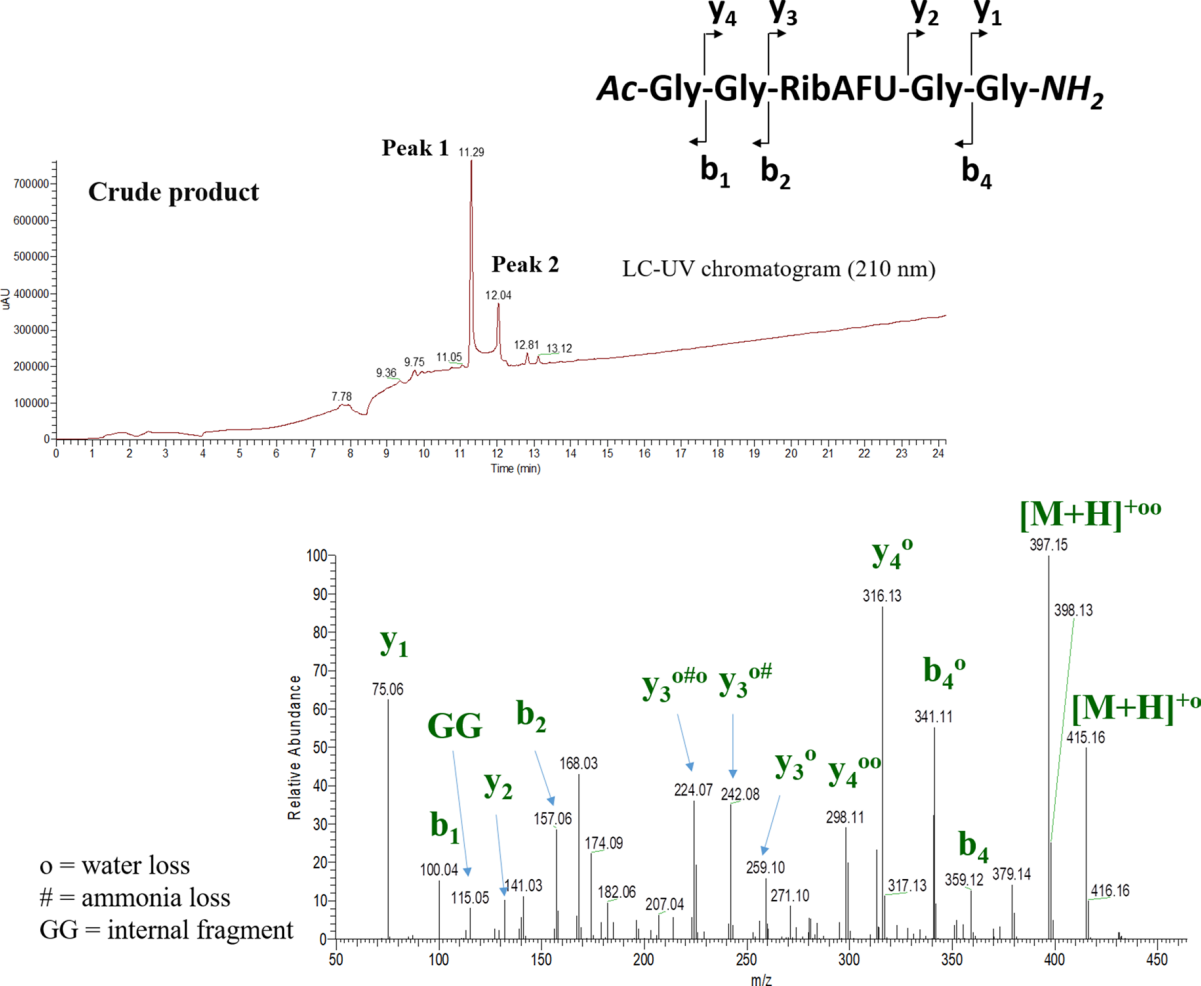
HILIC LC–UV–MS chromatogram of Ac-GG-RibAFU-GG-NH_2_ (**16**) and HCD MS/MS spectrum of its singly protonated compound acquired at 20 eV collision energy

Our ultimate goal was to apply the currently fine-tuned deprotection method for making various peptides and chimera sequences. The previously successfully applied -GG-**X**-GG- model system was synthesized here with X=RibAFU(ip) and conditions to remove its 1,2-*O*-isopropylidene protecting group were probed. The synthesis of -GG-RibAFU(ip)-GG- was carried out with Fmoc-strategy introducing Fmoc-RibAFU(ip)-OH (**8**) on RAM-Tentagel^®^ or 2-Cl-Trt-Cl resin (Scheme 3) by PyBOP/DIEA which was found as one of the most effective coupling reagents for SAAs (Nagy et al. 2019; Goldschmidt Gőz et al. 2019). The *N*-terminus of this model peptide were protected to avoid unwanted side-reactions (e.g. esterification). Peptide **18** was used for evaluation of peptide synthesis method and an intermediate of methyl glycoside formation. The deprotection of 1,2-*O*-isopropylidene moiety was carried out with the mixture of TFA/DCM/TIS/H_2_O which is in fact a common cleavage cocktail in SPPS. The 50% TFA condition was used to remove peptides from the resin and in parallel to complete the 1,2-*O*-isopropylidene deprotection (Scheme 3, step *e*). The removal of the 1,2-*O*-isopropylidene group gave successfully both pentapeptides with free 1,2-OHs (**16**, **17**). In the mixture of the crude product, 4:1/α:β anomeric ratio was observed at 11.29 min and 12.04 min, respectively (Fig. 5). The formation of methyl glycoside was accomplished after the final cleavage (Scheme 3, step *g* and *h*) using two routes. The Amberlite IR-120 H^+^ (8 eqv.)/MeOH condition was executed on compound **18** with 1,2-*O*-isopropylidene protection (Route **D**) and on compound **17** with fully unprotected SAA (Route **E**). In both cases, the Ac-GG-RibAFU(Me)-GG-OMe (**19**) pentapeptide was observed. However, Route **E** was faster for methyl glycoside formation, with retention times of 4.75 min and 5.54 min presenting a 4:1/α:β anomeric ratio, respectively (SFig. 19).

Although RP-HPLC was used to follow the deprotection reaction, there were components (**9**, **12**, **13**) and peptides (**16**, **17**, **19**) having a short retention time due to their increased hydrophilicity. The analysis of these polar compounds was a challenge, as their interaction with C-18 stationary phases was insufficient, leading to a very difficult product isolation. Products (**9**, **16**, **17**, **19**) were characterized via HILIC LC–UV–MS (Jablonski, Hudalla and Fountain 2012; Yoshida 2004) measurements with BEH Amide column capable of analyzing hydrophilic SAAs or sequences (Fig. 5). The optimized condition was found to enhance the retention of hydrophilic polypeptides, making analysis and isolation more adequate.

## Conclusions

A rather general 1,2-*O*-isopropylidene deprotection method was worked out for α/β-chimera peptides using a suitable d-*ribo* furanoid β-SAA model system, namely the N_3_-RibAFU(ip)-OH synthetic intermediate. To obtain the unprotected derivatives, two different conditions were studied successfully, namely (1) various TFA concentrations to form the free 1,2-OH product (**9**) and (2) various equivalents of Amberlite IR-120 H^+^ resin or TFA in MeOH forming methyl glycoside (**10**) to prevent the furanoid ring opening. In the first case, 50% TFA in DCM with TIS and H_2_O as scavengers was found to give the fully unprotected compound (N_3_-RibAFU-OH, **9**). Furthermore, 8 eqv. Amberlite IR-120 H^+^ resin in MeOH at 60 °C turned out to be optimum to furnish methyl glycoside methyl ester N_3_-RibAFU(Me)-OMe (**10**). These optimized methods, 50% TFA and H^+^ resin/MeOH, were applied successfully during the synthesis of α/β-chimera oligopeptide [-GG-RibAFU(ip)-GG-] to form the Ac-GG-RibAFU-GG-NH_2_ (**16**) without 1,2-*O*-isopropylidene protection and the methyl glycoside variant, namely the Ac-GG-RibAFU(Me)-GG-OMe (**19**). The described, fine-tuned conditions of deprotection were shown to be appropriate and sufficiently mild to remove 1,2-*O*-isopropylidene protection in oligo- and polypeptides of more complex amino acid sequences.

### Experimental section

#### Reagents and instrumentations

Reagents, materials and solvents were obtained from Sigma-Aldrich, Merck, Reanal and VWR. Moisture-sensitive solvents were dried on molecular sieve (3 Å). The capacity of Amberlite IR-120 H^+^ resin was 1.80 eq/L. Reactions were followed by RP-HPLC on a Phenomenex Jupiter C-18 column or Synergy™ 4 μm Hydro-RP 80 Å LC column with eluents 0.1% TFA in H_2_O (A) and 0.08% TFA, 95% acetonitrile/5% H_2_O (B), flow rate 1.0 ml/min and UV detection at 220 and 280 nm. Gradient were as follows: 0 min: 0% B, 30 min: 60% B, 32 min: 95% B, 33 min: 0% B, 45 min: 0% B and 45.1 min: 0% B for Phenomenex Jupiter C-18 column, and 0 min: 0% B, 30 min: 60% B, 33 min: 95% B, 39 min: 95% B, 40 min: 0% B, 45 min: 0% B and 45.2 min: 0% B for Synergy™ 4 μm Hydro-RP 80 Å LC column. MS spectra were performed with Bruker Esquire 3000 + tandem quadrupole mass spectrometer equipped with an electrospray ion source. FTIR spectra were recorded on a Bruker IFS 28 spectrometer by ATR technique. ^1^H NMR measurements were implemented with Bruker Avance 250 spectrometer in CDCl_3_ or DMSO-*d*_6_ at room temperature. Deuterated solvents were purchased from Eurisotop. Hydrophilic compounds were analyzed by HILIC LC–UV–MS approach. The measurements were executed on a column Waters Acquity BEH Amide UPLC (2.1 × 150 mm, 1.7 µm) using 20 mM ammonium acetate (A) and 100% acetonitrile (B) with flow rate 250 µl/min, UV detection at 210 and 280 nm and 40 °C column temperature. Dionex 3000 UHPLC was coupled to a Q Exactive Focus orbitrap mass spectrometer (Thermo Scientific, Bremen, Germany). ESI–MS spectra were acquired in *m/z* 200–800 (spray voltage: 3.5 kV; sheath gas: 46 au; aux. gas: 11 au; capillary temp: 360 °C; probe heater: 406 °C). MS/MS spectra were acquired using higher energy collision induced dissociation (HCD) at 20 eV. Gradients were as follows: 0 min: 90% B, 2 min: 90% B, 22 min: 40% B, 23 min: 40% B, 24 min: 90% B and 30 min: 90% B for peptides, and 0 min: 95% B, 2 min: 95% B, 22 min: 60% B, 23 min: 60% B, 24 min: 95% B and 30 min: 95% B for SAAs.

#### 1,2-*O*-Isopropylidene-3-azido-3-deoxy-α-d-ribofuranuronic acid (**7**)

Compound **7** was prepared based on previous results of our group (Nagy et al. 2017). RP-HPLC: 11.9 min or 21.6 min; FTIR-ATR: cm^−1^ *ν*_max_: 3500–2400 (OH), 2112 (N_3_), 1724 (C=O); ESI–MS: *m/z* calculated for C_8_H_11_N_3_O_5_ [M−H]^−^ 228.06205, found 228.06207; ^1^H NMR (DMSO-d_6_, 250 MHz) *δ* ppm 5.84 (d, *J *= 3.3 Hz, 1H), 4.78 (m, 1H), 4.35 (d, *J *= 9.7 Hz, 1H), 3.85 (dd, *J *= 9.6 and 4.5 Hz, 1H), 1.44 (s, 3H), 1.28 (s, 3H); HILIC LC–UV–MS: 2.13 min, *m/z* calculated for C_8_H_11_N_3_O_5_ [M−H]^−^ 228.06204, found 228.06094.

#### 1,2-*O*-Isopropylidene-*N*-(9-fluorenylmethoxy-carbonyl)-3-amino-3-deoxy-α-d-ribofuranuronic acid (**8**)

Compound **8** was prepared based on previous results of our group (Nagy et al. 2017). RP-HPLC: 21.0 min; FTIR-ATR: cm^−1^ *ν*_max_: 3500–2400 (OH), 3367 (NH), 1720 (C=O), 1692 (aromatic); ESI–MS: *m/z* calculated for C_23_H_23_NO_7_ [M+H]^+^ 426.2, found 426.2; ^1^H NMR (DMSO-d_6_, 250 MHz) *δ* ppm 7.89 (d, 2H), 7.69 (m, 3H), 7.36 (m, 5H), 5.85 (s, 1H), 4.61 (s, 1H), 4.29 (m, 3H), 4.01 (m, 1H), 1.46 (s, 3H), 1.26 (s, 3H).

#### *N*-Methyl-1,2-*O*-isopropylidene-3-azido-3-deoxy-α-d-ribofuranuronamide (**11**)

1,2-*O*-isopropylidene-3-azido-3-deoxy-α-d-ribofuranuronic acid (**7**, 200 mg; 0.87 mmol) was dissolved in DCM (49 ml), then CH_3_NH_2_·HCl (120 mg), EDC·HCl (671 mg), DIEA (0.6 ml) and HOBt (235 mg) were added. The mixture was stirred at room temperature for 3 h (EtOAc-Hex 2:1). After the reaction was complete, DCM (25 ml) was added to the mixture and extracted with 2 N HCl (2 × 50 ml), then washed with H_2_O (1 × 50 ml), saturated NaHCO_3_ solution (1 × 50 ml) and H_2_O (1 × 50 ml). The organic phase was dried (Na_2_SO_4_), filtered and concentrated in vacuo. The residue was treated by hexane to afford the product as white solid (160 mg, 76%). RP-HPLC: 9.9 min; ESI–MS: *m/z* calculated for C_9_H_14_N_4_O_4_ [M+H]^+^ 243.10933 and [M+Na]^+^ 265.09128, found 243.10859 and 265.09035, respectively; ^1^H NMR (CDCl_3_, 250 MHz) *δ* ppm 6.43 (s, 1H), 5.84 (d, *J *= 3.3 Hz, 1H), 4.71 (t, *J *= 3.6 Hz, 1H), 4.48 (d, *J *= 9.5 Hz, 1H), 3.65 (dd, *J *= 9.3 and 4.3 Hz, 1H), 2.86 (d, *J *= 4.9 Hz, 3H), 1.57 (s, 3H), 1.37 (s, 3H).

#### The removal of 1,2-*O*-isopropylidene protection from the azido derivative (7)

##### 3-Azido-3-deoxy-d-ribofuranuronic acid (**9**)

(A) 1,2-*O*-Isopropylidene-3-azido-3-deoxy-α-d-ribofuranuronic acid (**7**, 100 mg; 0.44 mmol) was dissolved in 5 ml of different concentrations of TFA (50%, 70%) in DCM (45% and 25%, respectively) with TIS (2.5%) and H_2_O (2.5%). The mixtures were stirred at room temperature for 3 h and evaporated in vacuo. The residues were dissolved in 1,2-dimethoxyethane (5 ml) and concentrated to obtain white oils in both cases. RP-HPLC: 4.3 min; ESI–MS: *m/z* calculated for C_5_H_7_N_3_O_5_ [M−H]^−^ 188.03075, found 188.03029; HILIC LC–UV–MS: 10.46 min, *m/z* calculated for C_5_H_7_N_3_O_5_ [M−H]^−^ 188.03074, found 188.03057.

(B) 1,2-*O*-Isopropylidene-3-azido-3-deoxy-α-d-ribofuranuronic acid (**7**, 50 mg; 0.22 mmol) was added into 2.5 ml mixtures of TFA (50%, 70%, 90%) in DCM (45%, 25%, 5%, respectively), TIS (2.5%) and H_2_O (2.5%). Reactions were stirred at room temperature. Fractions of reaction mixtures were concentrated by air blowing, diluted in water then measured by RP-HPLC (SFig. 22, SFig. 23, SFig. 24).

##### Methyl 3-azido-3-deoxy-d-ribofuranuronate methyl ester (**10**)

(A) 1,2-*O*-Isopropylidene-3-azido-3-deoxy-α-d-ribofuranuronic acid (**7**, 50 mg; 0.22 mmol) was dissolved in dried MeOH (2 ml) and Amberlite IR-120 H^+^ resin (2, 4, 8 and 12 eqv. corresponding to 100, 200, 400 or 600 mg) were added. The mixtures were heated to 60 °C for 6 h. The reactions were followed by RP-HPLC (SFig. 20, SFig. 21). The mixtures were filtered and washed with MeOH. Filtrates were evaporated in vacuo to obtain oil products. The mixture of products containing mainly the final product **10** (~ 90%) was characterized. RP-HPLC: 9.0 min; ESI–MS: *m/z* calculated for C_7_H_11_N_3_O_5_ [M+Na]^+^ 240.1, found 240.1; ^1^H NMR (CDCl_3_, 250 MHz) *δ* ppm 4.93 (s, 1H), 4.58 (d, *J *= 7.2 Hz, 1H), 4.36 (m, 1H), 4.16 (d, *J *= 4.4 Hz, 1H), 3.83 (s, 3H), 3.42 (s, 3H).

(B) 1,2-*O*-Isopropylidene-3-azido-3-deoxy-α-d-ribofuranuronic acid (**7**, 50 mg; 0.22 mmol) was dissolved in dried MeOH (2 ml) and Amberlite IR-120 H^+^ resin (400 mg, 8 eqv.) was added to the solution. The reaction mixture was stirred at different temperatures (60 °C, 40 °C and RT). Fractions of reaction mixtures were neutralized (NaHCO_3_), centrifuged, diluted in MeOH then measured by RP-HPLC (STable 1, STable 2).

(C) 1,2-*O*-Isopropylidene-3-azido-3-deoxy-α-d-ribofuranuronic acid (**7**, 50 mg; 0.22 mmol) was added into 2 ml mixtures of TFA (30%, 50%, 70%) and dried MeOH (70%, 50%, 30%, respectively). Reactions were stirred at room temperature overnight and evaporated in vacuo to obtain oil product. Mixtures were measured by RP-HPLC (SFig. 27).

#### The removal of 1,2-*O*-isopropylidene protection from methylamide derivative (11)

##### *N*-Methyl-3-azido-3-deoxy-d-ribofuranuronamide (12)

Compound **11** (40 mg; 0.17 mmol) was dissolved in the mixture (2 ml) of TFA (50%), DCM (45%), TIS (2.5%) and H_2_O (2.5%) and stirred at room temperature for 1 h. The reaction was monitored by TLC (EtOAc-Hex 2:1) and RP-HPLC (SFig. 26). The solvent was removed in vacuo and the residue was treated with cold Et_2_O to precipitate the solid product. ESI–MS: *m/z* calculated for C_6_H_10_N_4_O_4_ [M+H]^+^ 203.07803, found 203.07722.

##### Methyl *N*-methyl-3-azido-3-deoxy-d-ribofuranosiduronamide (13)

Compound **11** (40 mg; 0.17 mmol) was dissolved in dried MeOH (1.5 ml) and Amberlite IR-120 H^+^ resin (300 mg, 8 eqv.) was added to the solution. The reaction was stirred at 60 °C for 6 h, monitored by TLC (EtOAc-Hex 2:1) and RP-HPLC. The mixture was filtered and washed with MeOH. The filtrate was concentrated in vacuo to achieve the oil product. ESI–MS: *m/z* calculated for C_7_H_12_N_4_O_4_ [M+Na]^+^ 239.07563 and [M+H]^+^ 217.09368, found [M+Na]^+^ 239.07487 and [M+H]^+^ 217.09303, respectively (SFig. 25).

##### Model peptide

SPPS of the model peptide was executed manually on RAM-Tentagel^®^ or 2-Cl-Trt-Cl resin with the standard methodology using Fmoc-strategy. Resins were swollen in DCM. Coupling of Fmoc-Gly-Gly-OH to resins was implemented in two different methods. For RAM-Tentagel^®^ resin, the first step was the removal of Fmoc-group conducted with 2% piperidine and 2% DBU in DMF (10 + 20 min). The coupling was accomplished using Fmoc-Gly-Gly-OH (3 eqv. to the nominal capacity of the resin ~ 0.24 mmol/g) dissolved in DMF and PyBOP (3 eqv.)/DIEA (6 eqv.) added to the solution. In the case of 2-Cl-Trt-Cl resin, the coupling was made with Fmoc-Gly-Gly-OH (1.5 eqv. to the nominal capacity of the resin ~ 1.60 mmol/g to tune down to 0.28 mmol/g) which was dissolved in DMF and DIEA (3.75 eqv.) was added to the solution. Afterwards, amino acids were coupled to resins using reagent pairs PyBOP/DIEA in DMF. Coupling of amino acids lasted for 1 h, whereas that of Fmoc-RibAFU(ip)-OH finished in 3 h. After coupling, resins were washed with 3 × DMF, 3 × DCM, 2 × MeOH, 1 × Et_2_O and dried in vacuo. The capacity of the resin was determined by spectrometric measurement of the amount of Fmoc chromophore (Fmoc-piperidine adduct) released upon treatment of the resin with 50% piperidine in DMF (Chan and White 2000). Fmoc-deprotection was done by 2% piperidine and 2% DBU in DMF (3 + 17 min). The successful removal was analyzed by Kaiser test. The acetylation was performed with Ac_2_O:DIEA:DMF (1:1.2:3) for 30 min.

##### Ac-Gly-Gly-RibAFU-Gly-Gly-NH_2_ (16)

The peptide was cleaved from RAM-Tentagel^®^ resin (50 mg) with TFA (50%), DCM (45%), TIS (2.5%) and H_2_O (2.5%) for 3 h. The resin was washed with 3 × DCM and 3 × MeOH, then solvent was removed in vacuo. The residue was treated with cold Et_2_O to precipitate white solid product (4.9 mg). HILIC LC–UV–MS: 11.29 min and 12.04 min, *m/z* calculated for C_15_H_24_N_6_O_9_ [M+H]^+^ 433.1683 and [M+Na]^+^ 455.1502, found 433.1676 and 455.1493, respectively.

##### Ac-Gly-Gly-RibAFU-Gly-Gly-OH (17)

The peptide was cleaved from 2-Cl-Trt-Cl resin (150 mg) by a mixture of TFA (50%), DCM (45%), TIS (2.5%) and H_2_O (2.5%) for 3 h. The resin was washed with 3 × DCM and 3 × MeOH, then solvent was removed in vacuo. By treating the residue with cold Et_2_O, the white solid product was precipitated (14.1 mg). HILIC LC–UV–MS: 12.68 min and 12.94 min, *m/z* calculated for C_15_H_23_N_5_O_10_ [M+H]^+^ 434.15232, [M+Na]^+^ 456.13426 and [M+H–H_2_O]^+^ 416.14176, found 434.15109, 456.413304 and 416.14060, respectively.

##### Ac-Gly-Gly-RibAFU(ip)-Gly-Gly-OH (18)

The peptide was cleaved from 2-Cl-Trt-Cl resin (150 mg) with AcOH:MeOH:DCM (1:1:8) for 3 h. The resin was washed with 3 × DCM, 3 × iPrOH and 1 × Et_2_O. The solvent was removed in vacuo. The residue was precipitated in cold Et_2_O to obtain white solid product (11.4 mg, 58%). HILIC LC–UV–MS: 9.91 min, *m/z* calculated for C_18_H_27_N_5_O_10_ [M+H]^+^ 474.18362, found 474.18276.

##### Ac-Gly-Gly-RibAFU(Me)-Gly-Gly-OMe (19)

(A) Peptide **17** (13 mg) was dissolved in dried MeOH with Amberlite IR-120 H^+^ (8 eqv.). The mixture was stirred at 60 °C for 3 h, filtered and washed with MeOH. The filtrate was concentrated in vacuo. The product as white solid was precipitated with cold Et_2_O (7.8 mg). HILIC LC–UV–MS: 4.75 min and 5.54 min, *m/z* calculated for C_17_H_27_N_5_O_10_ [M+H]^+^ 462.18362 and [M+H–CH_3_OH]^+^ 430.15741, found 462.18214 and 430.15641, respectively.

(B) Peptide **18** (10 mg) was dissolved in dried MeOH with Amberlite IR-120 H^+^ (8 eqv.). The mixture was stirred at 60 °C for 6 h, filtered and washed with MeOH. The filtrate was concentrated in vacuo and the residue was treated in cold Et_2_O to obtain white solid product (6.7 mg).

## Electronic supplementary material

Below is the link to the electronic supplementary material.

Supplementary material 1 (PDF 2462 kb)

## Abbreviations

ACPC: 2-Aminocyclopentanecarboxylic acid
ACHC: 2-Aminocyclohexanecarboxylic acid
β-SAA: β-Sugar amino acid
SPPS: Solid-phase peptide synthesis
H-XylAFU(ip)-OH: 1,2-*O*-Isopropylidene-3-amino-3-deoxy-α-d-xylofuranuronic acid
H-XylAFU-OH: 3-Amino-3-deoxy-α-d-xylofuranuronic acid
H-RibAFU(ip)-OH: 1,2-*O*-Isopropylidene-3-amino-3-deoxy-α-d-ribofuranuronic acid
H-RibAFU-OH: 3-Amino-3-deoxy-α-d-ribofuranuronic acid
N_3_-RibAFU(ip)-OH: 1,2-*O*-Isopropylidene-3-azido-3-deoxy-α-d-ribofuranuronic acid
Fmoc-RibAFU(ip)-OH: 1,2-*O*-Isopropylidene-*N*-(9-fluorenylmethoxy-carbonyl)-3-amino-3-deoxy-α-d-ribofuranuronic acid
N_3_-RibAFU(ip)-NHMe: *N*-Methyl-1,2-*O*-isopropylidene-3-azido-3-deoxy-d-ribofuranuronamide
N_3_-RibAFU-OH: 3-Azido-3-deoxy-α-d-ribofuranuronic acid
N_3_-RibAFU-NHMe: *N*-Methyl-3-azido-3-deoxy-d-ribofuranuronamide
N_3_-RibAFU(Me)-OMe: Methyl 3-azido-3-deoxy-d-ribofuranuronate methyl ester
N_3_-RibAFU(Me)-NHMe: Methyl *N*-methyl-3-azido-3-deoxy-d-ribofuranosiduronamide
