# Supplementary material for: Synthesis of chimera oligopeptide including furanoid β-sugar amino acid derivatives with free OHs: mild but successful removal of the 1,2-O-isopropylidene from the building block
Source: Amino Acids. 2021 Feb 9;53(2):281–94. doi: 10.1007/s00726-020-02923-3 (PMC7910362; doi:10.1007/s00726-020-02923-3)
Supplement: Supplementary file 1 — Supplementary material 1 (PDF 2462 kb) [file 726_2020_2923_MOESM1_ESM.pdf]

Synthesis of chimera oligopeptide including furanoid β-sugar amino acid derivatives with free OHs: mild but successful removal of the 1,2-*O*-isopropylidene from the building block

Kim Hoang Yen Duong,[1] Viktória Goldschmidt Gőz,[2] István Pintér,[1] and András Perczel[*1,2]

[1] Laboratory of Structural Chemistry and Biology, Institute of Chemistry, ELTE Eötvös Loránd University - Pázmány P. stny. 1/A, 1117 Budapest, Hungary
[2] MTA-ELTE Protein Modeling Research Group, ELTE Eötvös Loránd University - Pázmány P. stny. 1/A, 1117 Budapest, Hungary

* *e-mail: perczel.andras@ttk.elte.hu*

Table of contents

NMR spectra of products 2
ESI-MS spectra of products 5
FTIR-ATR spectra of sugar amino acid derivatives 8
HILIC LC-UV-MS chromatograms of products 9
RP-HPLC chromatograms for following deprotection 15
Mechanism for deprotection components 19
Tables 20

NMR spectra of products

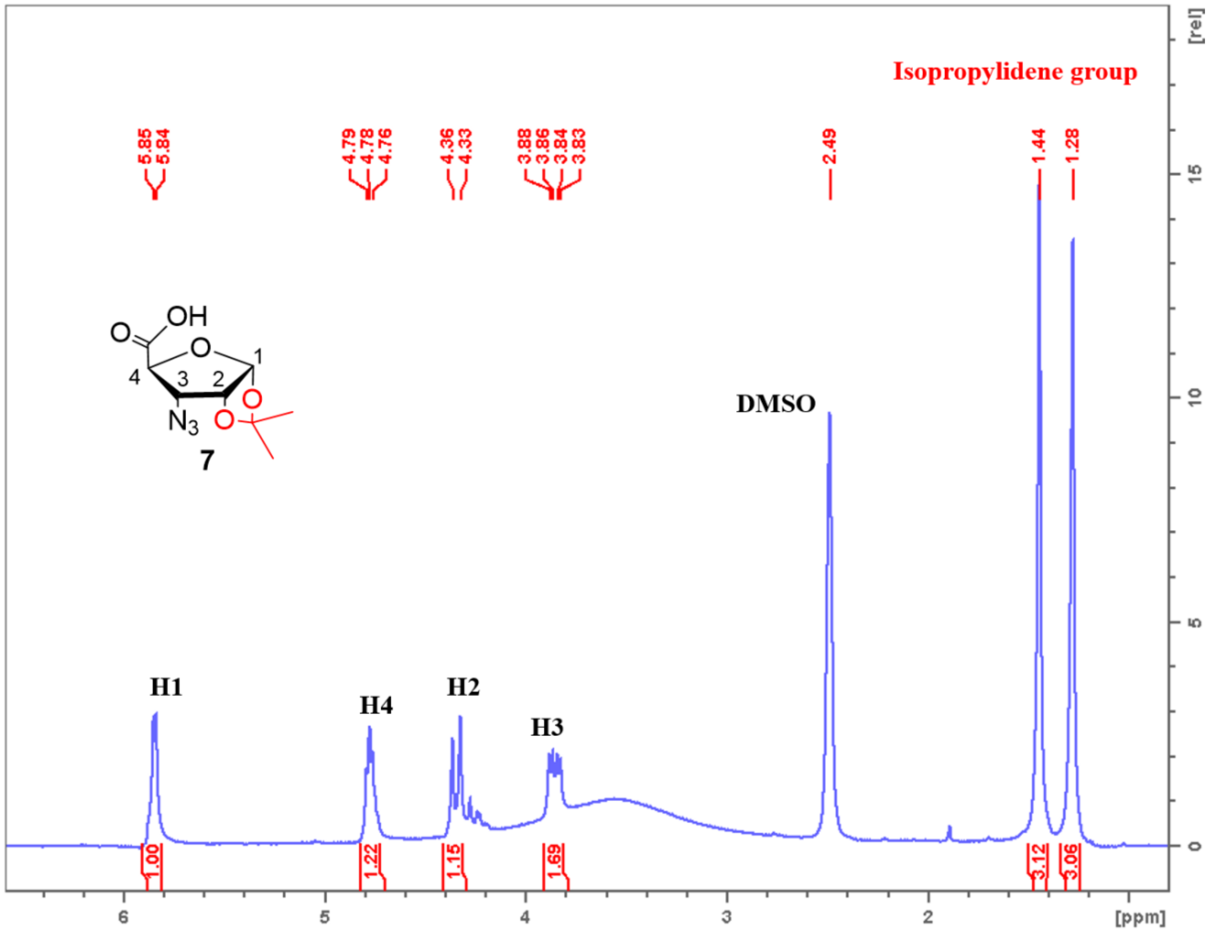

SFig. 1 ^{1}H NMR (250 MHz, DMSO-d$_6$, 25 °C) spectrum of model N$_3$-RibAFU(ip)-OH (**7**). The two signals of the 1,2-*O*-isopropylidene group are observed at 1.44 and 1.28 ppm

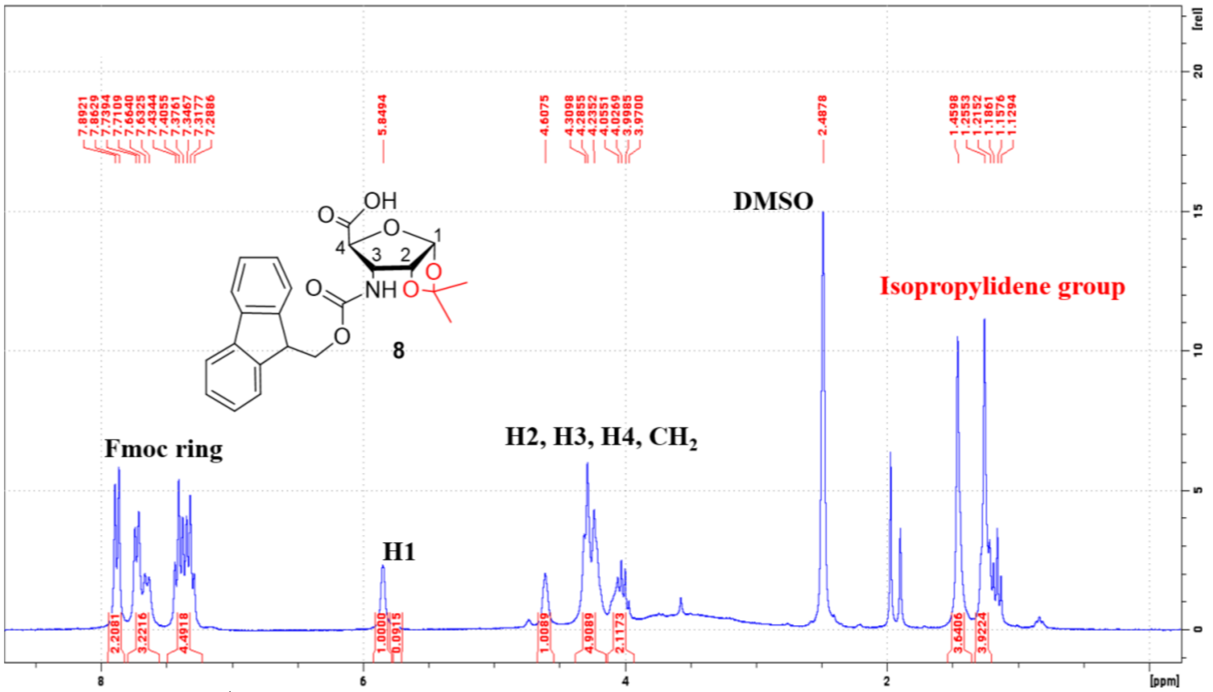

SFig. 2 ^{1}H NMR (250 MHz, DMSO-d$_6$, 25 ºC) spectrum of Fmoc-RibAFU(ip)-OH (**8**) sugar amino acid

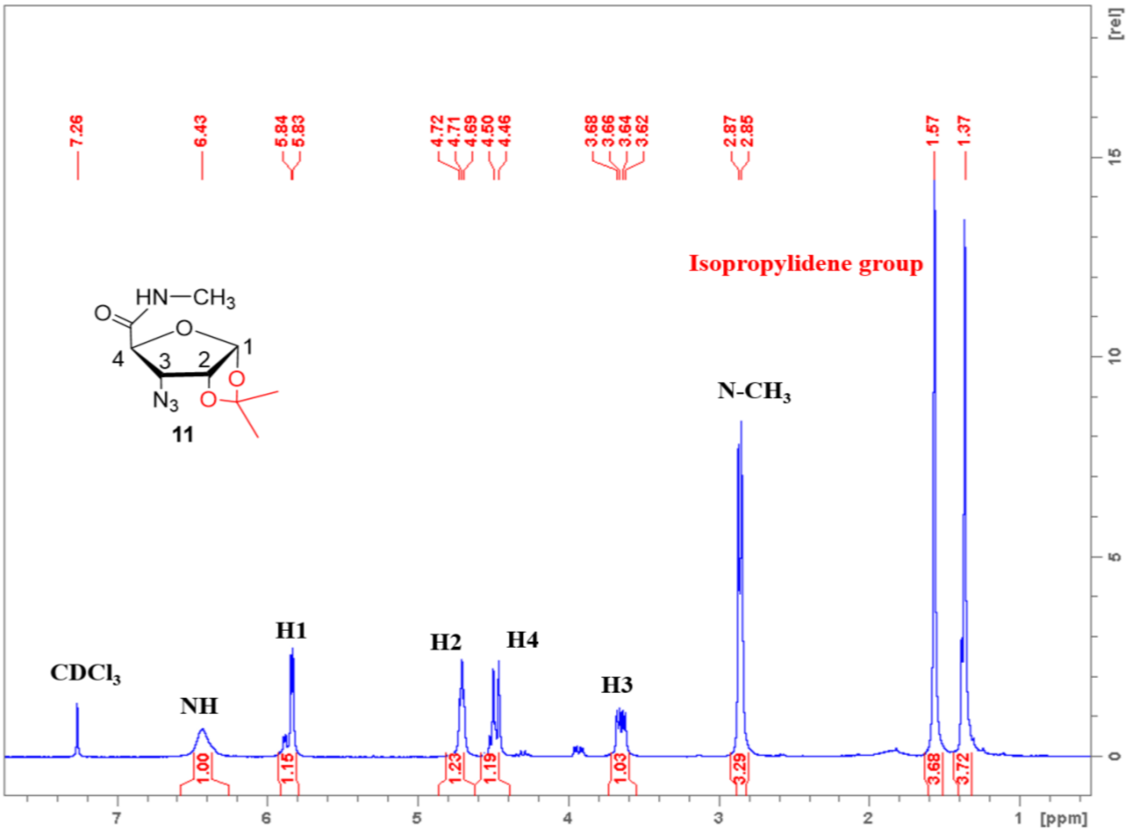

SFig. 3 ^{1}H NMR (250 MHz, $CDCl_3$, 25 °C) spectrum of N_3-RibAFU(ip)-NHMe (**11**). The two signals of the 1,2-*O*-isopropylidene group are observed at 1.57 and 1.37 ppm

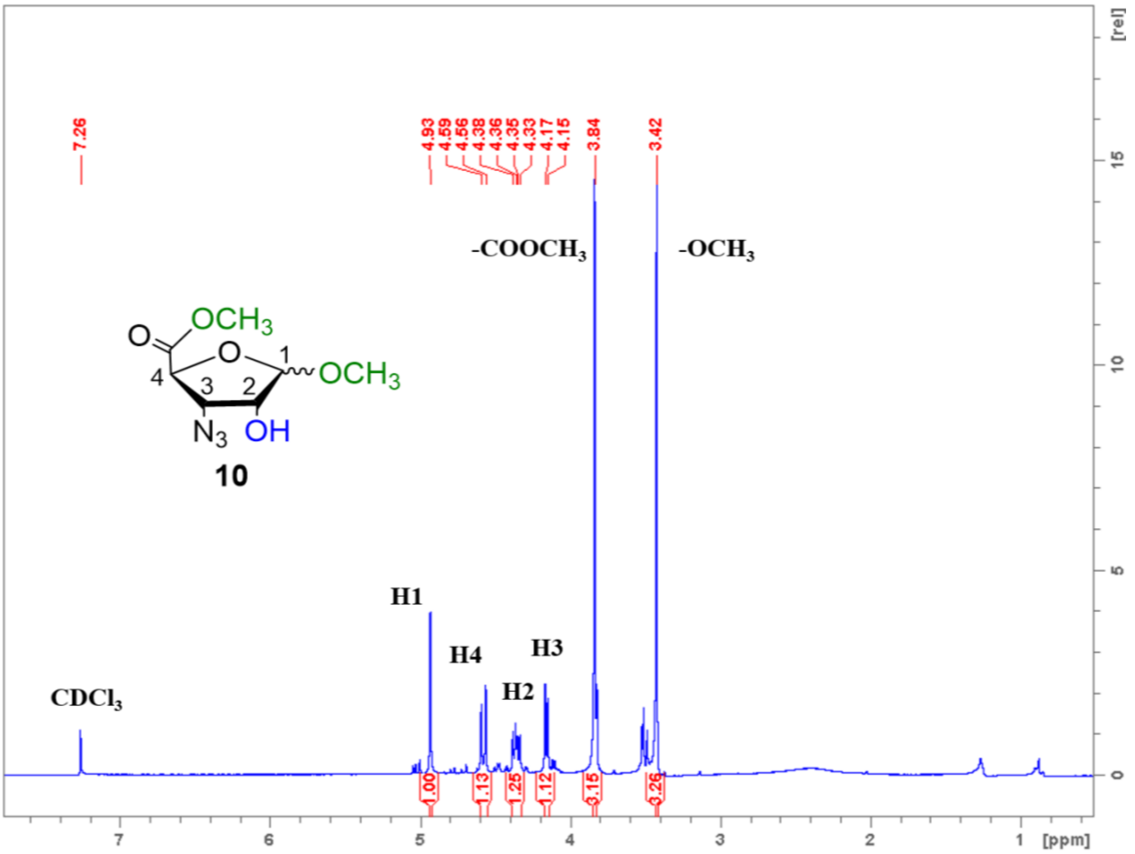

SFig. 4 ^{1}H NMR (250 MHz, $CDCl_3$, 25 °C) spectrum of N_3-RibAFU(Me)-OMe (**10**). The characteristic methyl signals of the methyl ester and the methyl *O*-glycoside are observed at 3.84 and 3.42 ppm, respectively. The main product is the α-anomer and the other by-product is the β-anomer, shown by ^{3}J couplings of H1-H2: < 1.0 Hz and 11.5 Hz, respectively

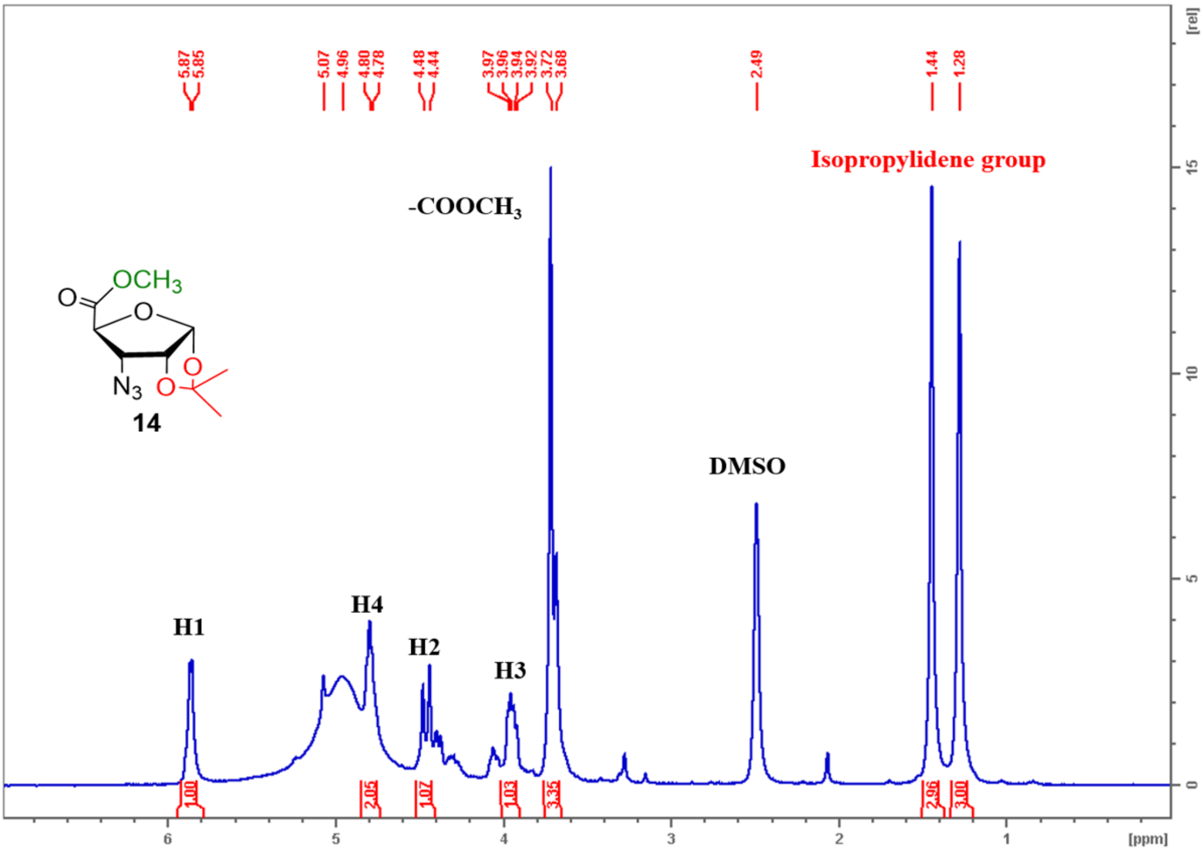

SFig. 5 ^{1}H NMR (250 MHz, DMSO-d$_6$, 25 °C) spectrum of N$_3$-RibAFU(ip)-OMe (**14**). The two signals of the 1,2-*O*-isopropylidene group are observed at 1.44 and 1.28 ppm

ESI-MS spectra of products

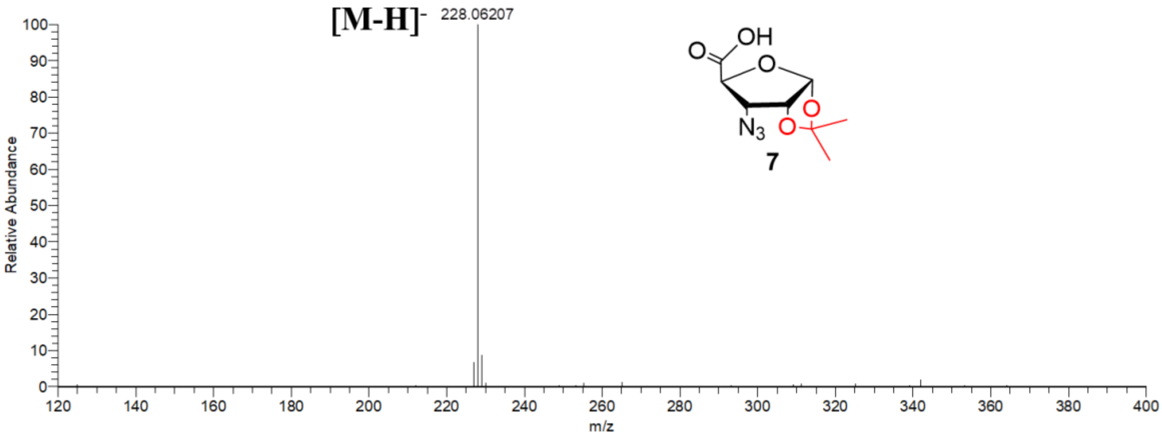

SFig. 6 HRMS spectrum of N_3-RibAFU(ip)-OH (**7**); *m/z* calculated for $C_8H_{11}N_3O_5$ $[M\text{-}H]^-$ 228.06205, found 228.06207

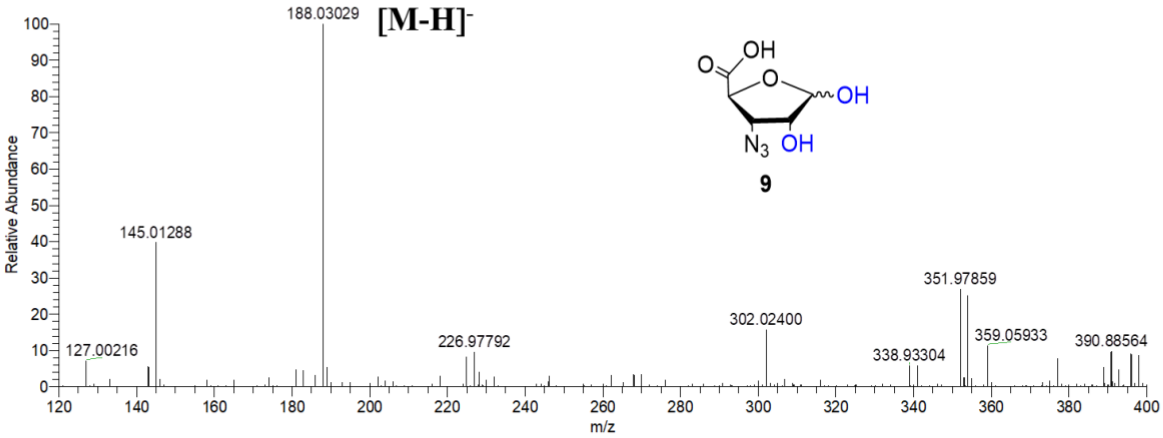

SFig. 7 HRMS spectrum of the crude product of the 1,2-*O*-isopropylidene removal from N_3-RibAFU(ip)-OH (**7**) with 50% TFA. The main product N_3-RibAFU-OH (**9**) could not be isolated in its pure form. HRMS: *m/z* calculated for $C_5H_7N_3O_5$ $[M\text{-}H]^-$ 188.03075, found 188.03029

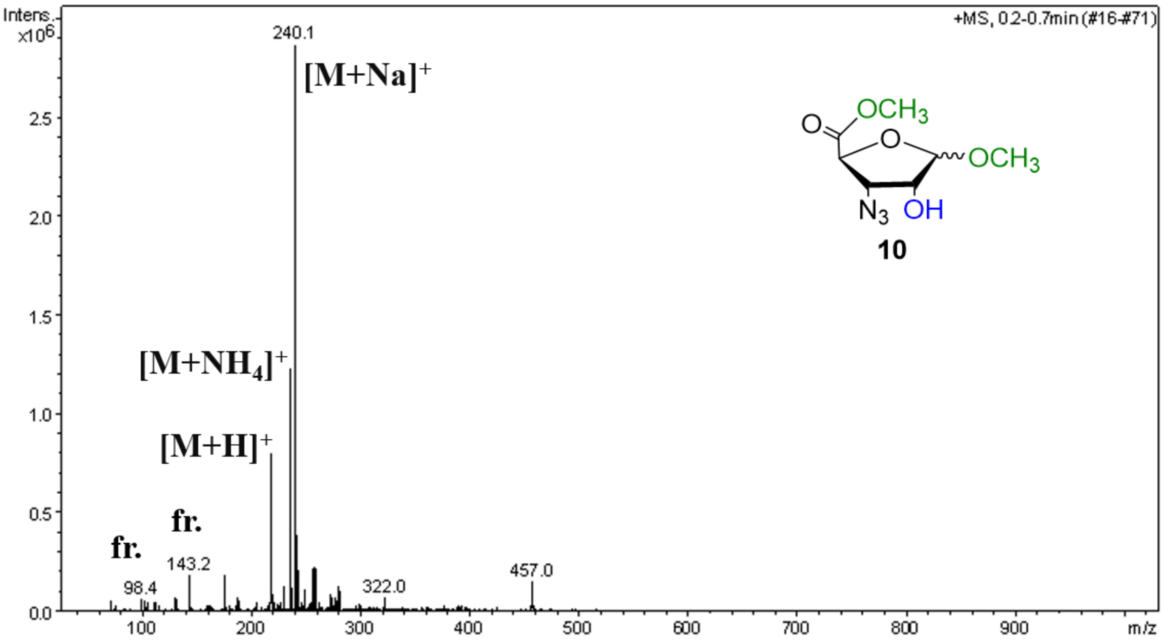

SFig. 8 MS spectrum of N_3-RibAFU(Me)-OMe (**10**); *m/z* calculated for $C_7H_{11}N_3O_5$ $[M+Na]^+$ 240.1, found 240.1

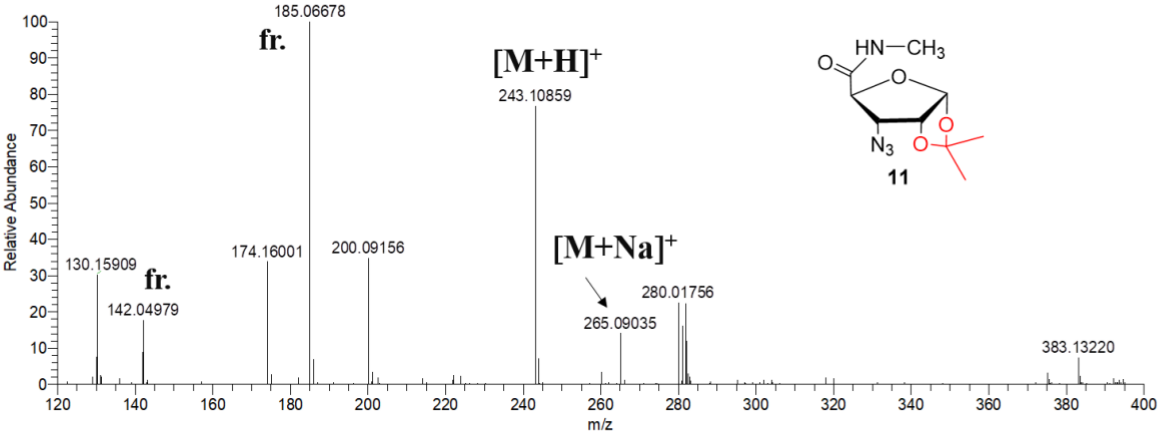

SFig. 9 HRMS spectrum of N_3-RibAFU(ip)-NHMe (**11**); *m/z* calculated for $C_9H_{14}N_4O_4$ $[M+H]^+$ 243.10933 and $[M+Na]^+$ 265.09128, found 243.10859 and 265.09035, respectively

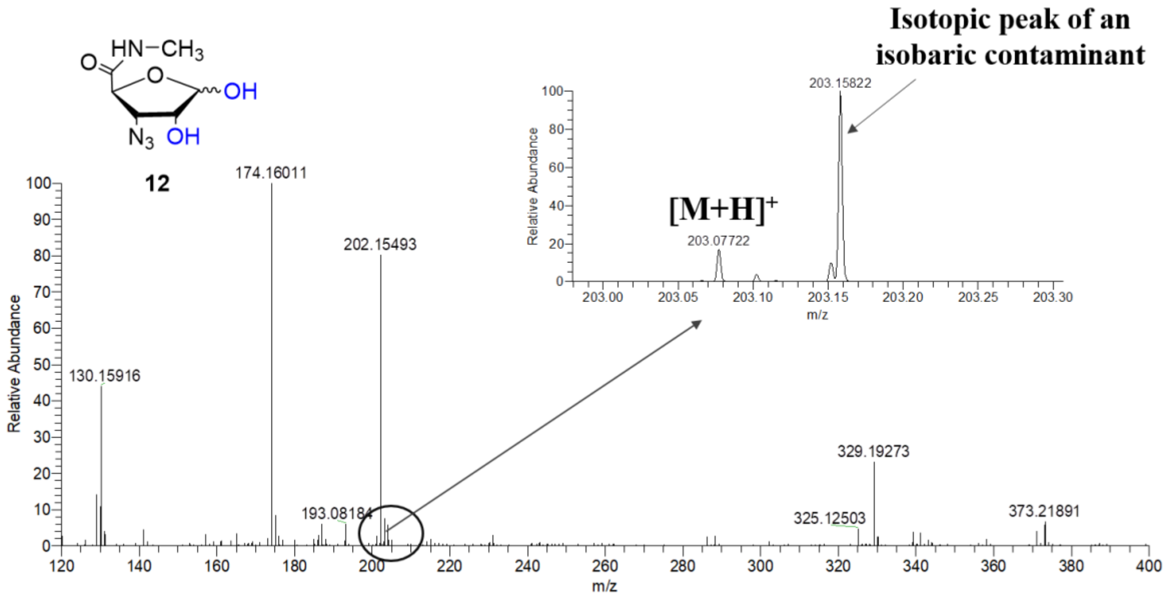

SFig. 10 HRMS spectrum of N_3-RibAFU-NHMe (**12**); *m/z* calculated for $C_6H_{10}N_4O_4$ $[M+H]^+$ 203.07803, found 203.07722

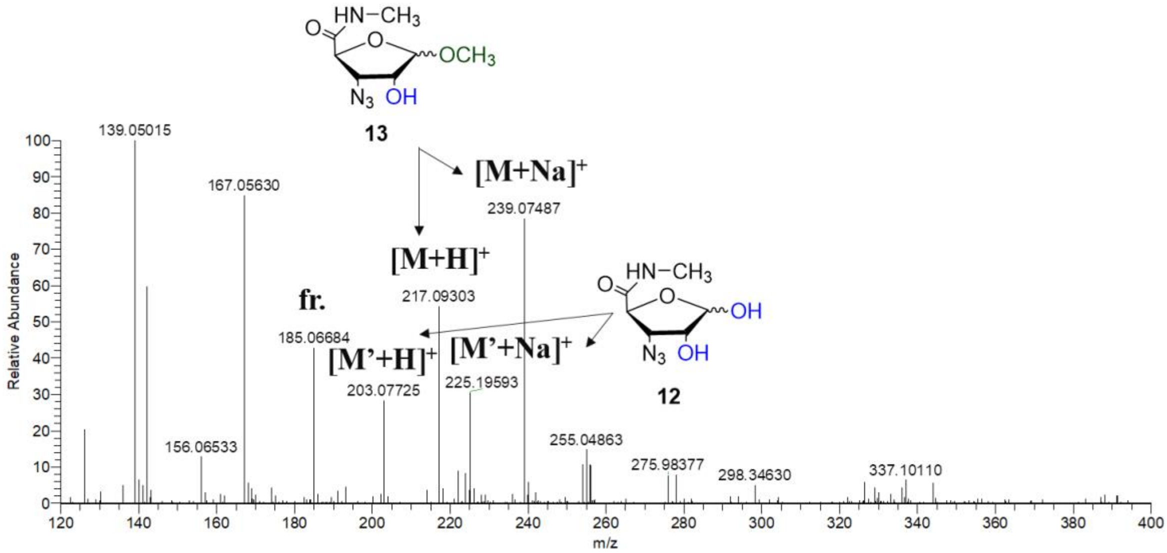

SFig. 11 HRMS spectrum of N_3-RibAFU(Me)-NHMe (**13**) in the mixture of compound **12** and **13** of the 1,2-*O*-isopropylidene removal from N_3-RibAFU(ip)-NMe (**11**) with Amberlite IR-120 H^+ resin (8 eqv.)/MeOH at 60 ºC; *m/z* calculated for $C_7H_{12}N_4O_4$ $[M+Na]^+$ 239.07563 and $[M+H]^+$ 217.09368, found $[M+Na]^+$ 239.07487 and $[M+H]^+$ 217.09303, respectively

FTIR-ATR spectra of sugar amino acid derivatives

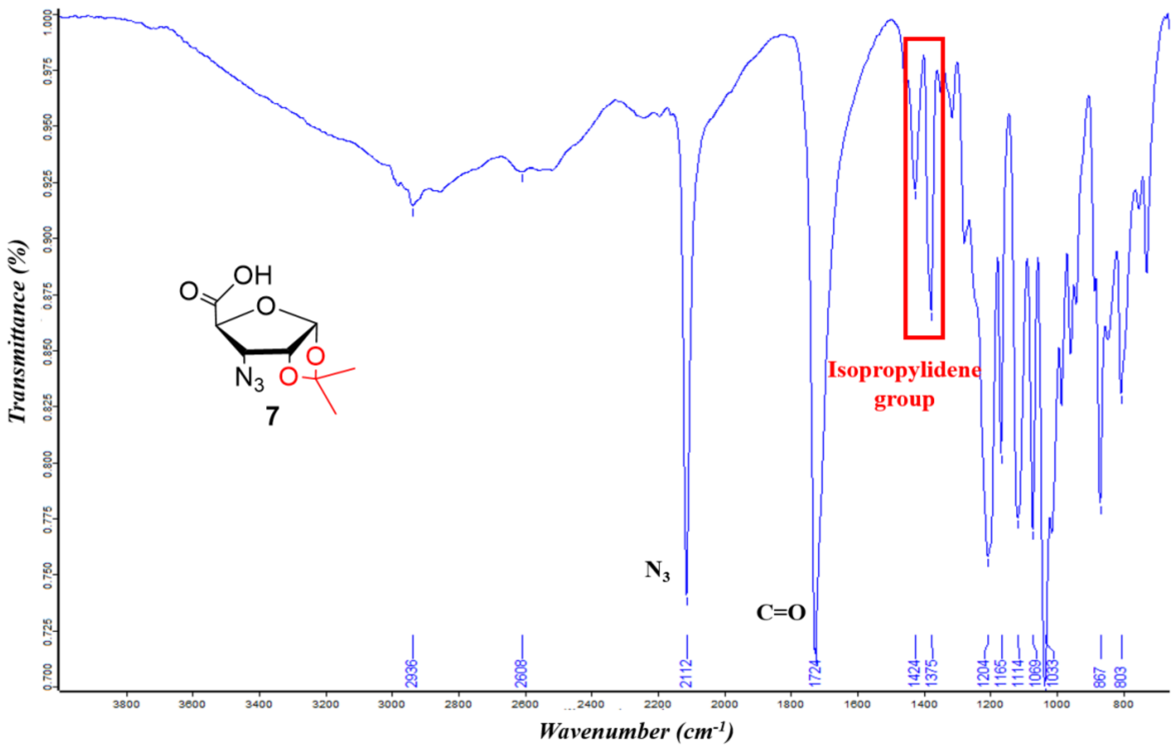

SFig. 12 FTIR-ATR spectrum of N_3-RibAFU(ip)-OH (**7**)

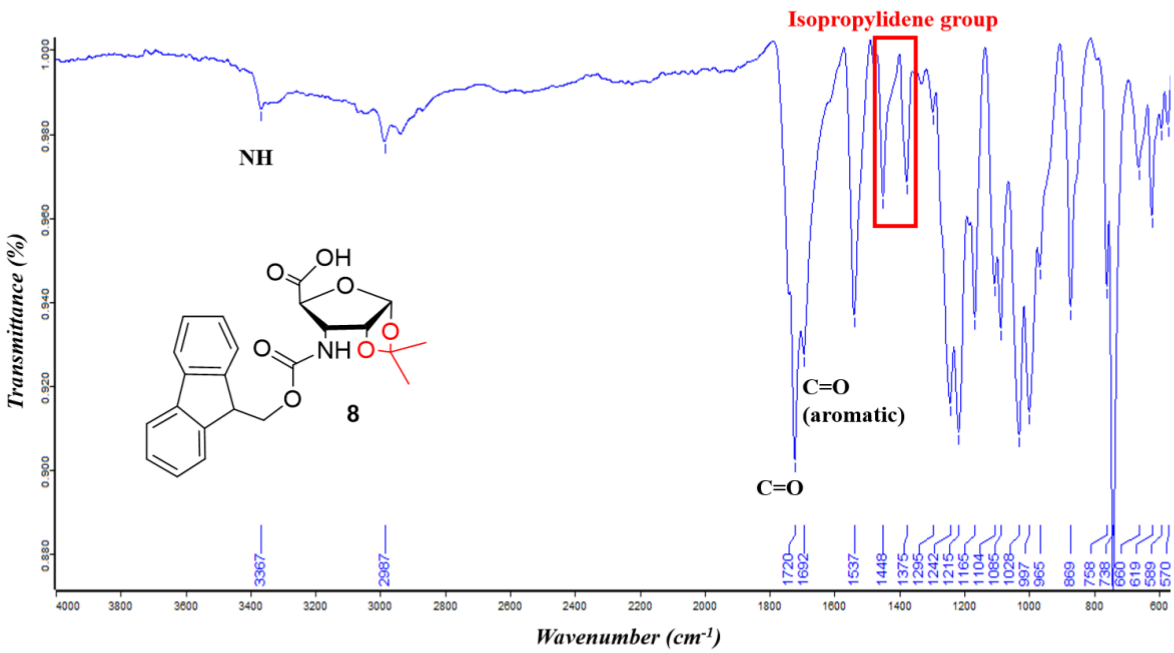

SFig. 13 FTIR-ATR spectrum of Fmoc-RibAFU(ip)-OH (**8**)

HILIC LC-UV-MS chromatograms of products

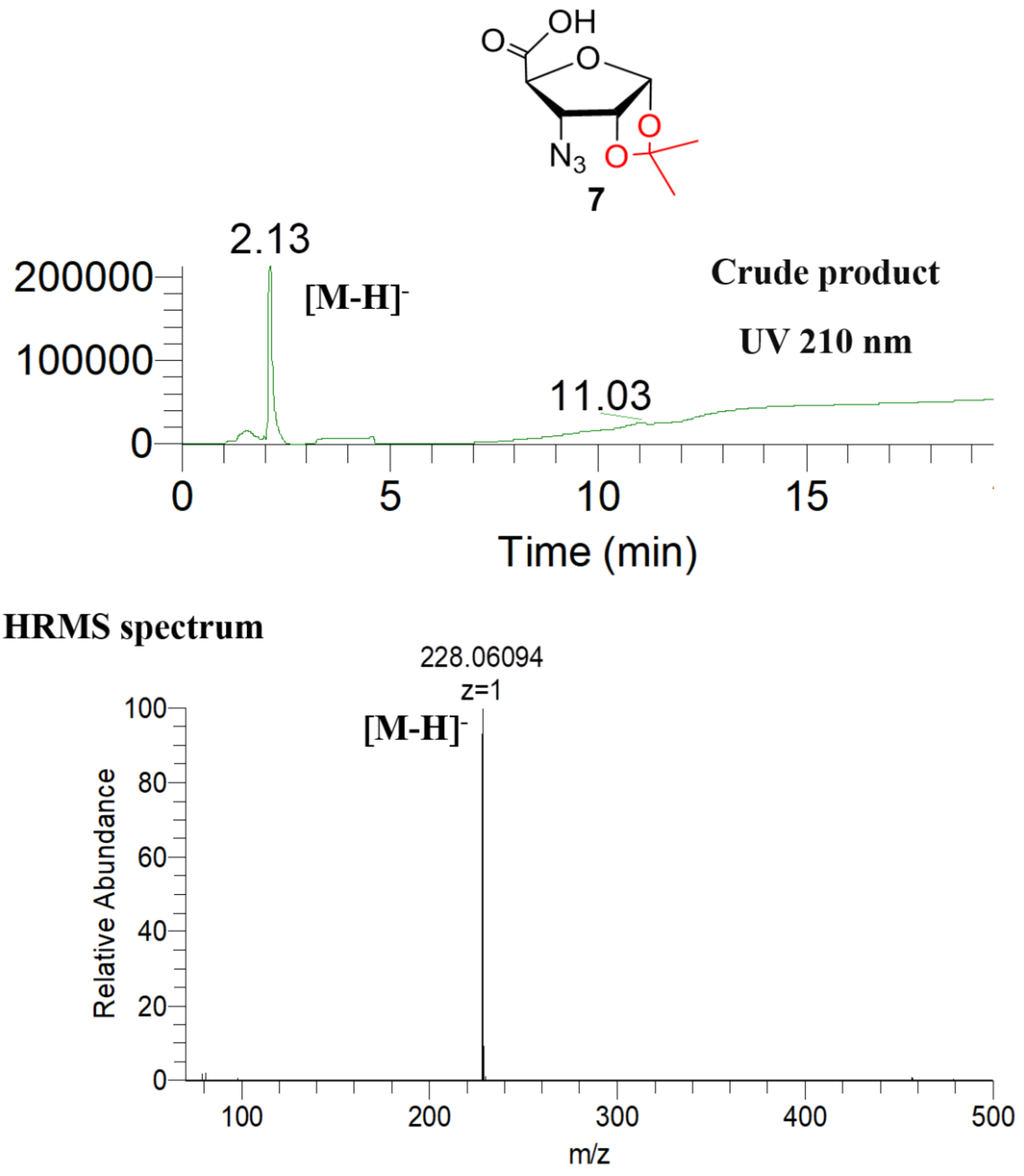

SFig. 14 HILIC LC-UV-MS chromatogram of N_3-RibAFU(ip)-OH (**7**). Retention time: 2.13 min; *m/z* calculated for $C_8H_{11}N_3O_5$ $[M-H]^-$ 228.06204, found 228.06094

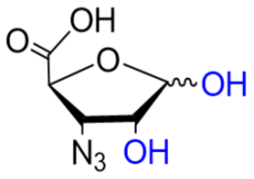

9

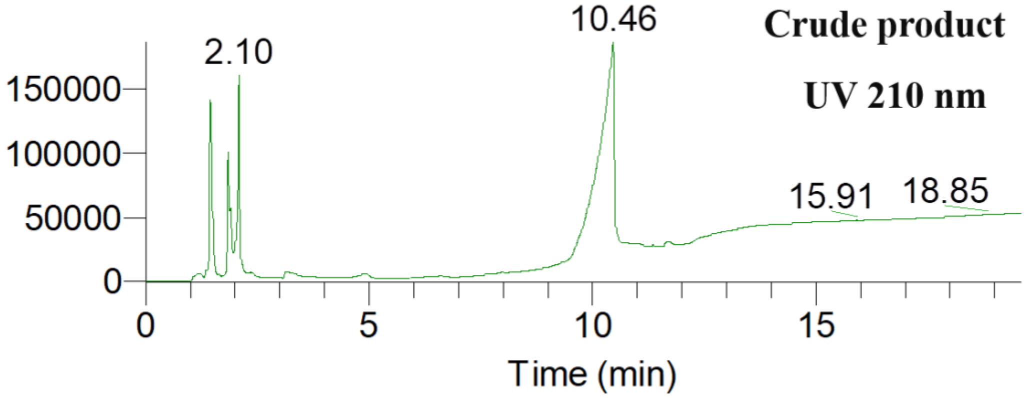

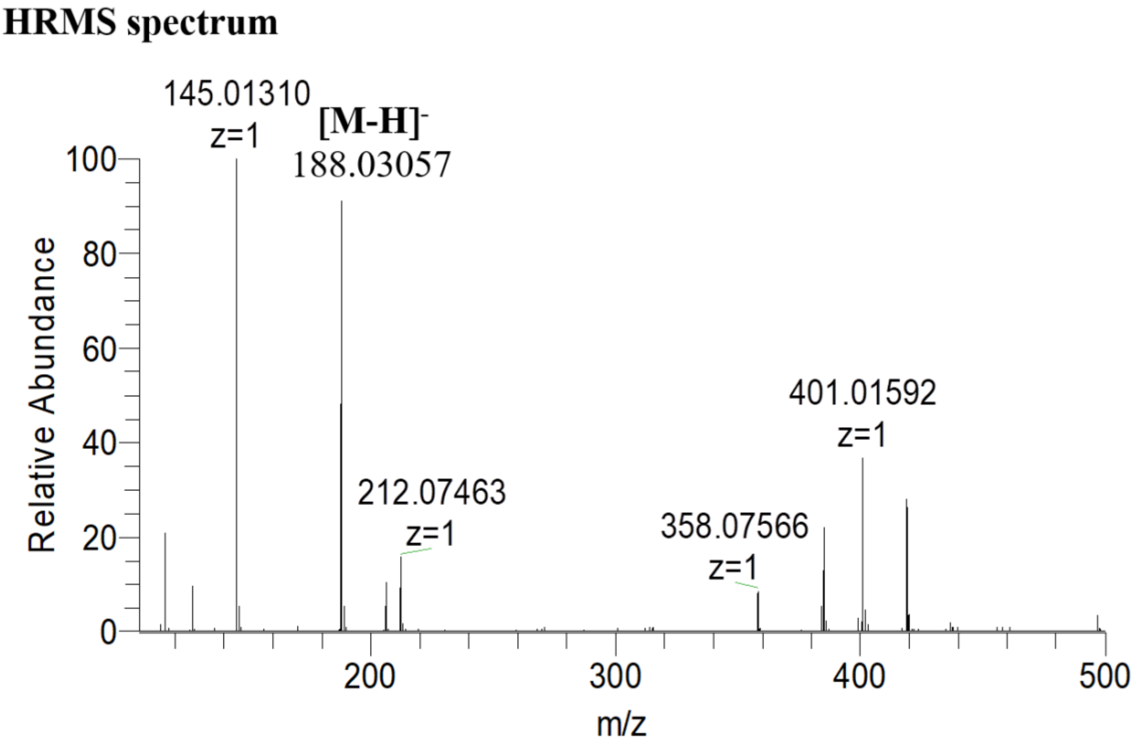

SFig. 15 HILIC LC-UV-MS chromatogram of the crude product of the 1,2-*O*-isopropylidene removal from N_3-RibAFU(ip)-OH (**7**) with 50% TFA. The product, N_3-RibAFU-OH (**9**), could be shown with retention time 10.46 min; *m/z* calculated for $C_5H_7N_3O_5$ $[M-H]^-$ 188.03074, found 188.03057

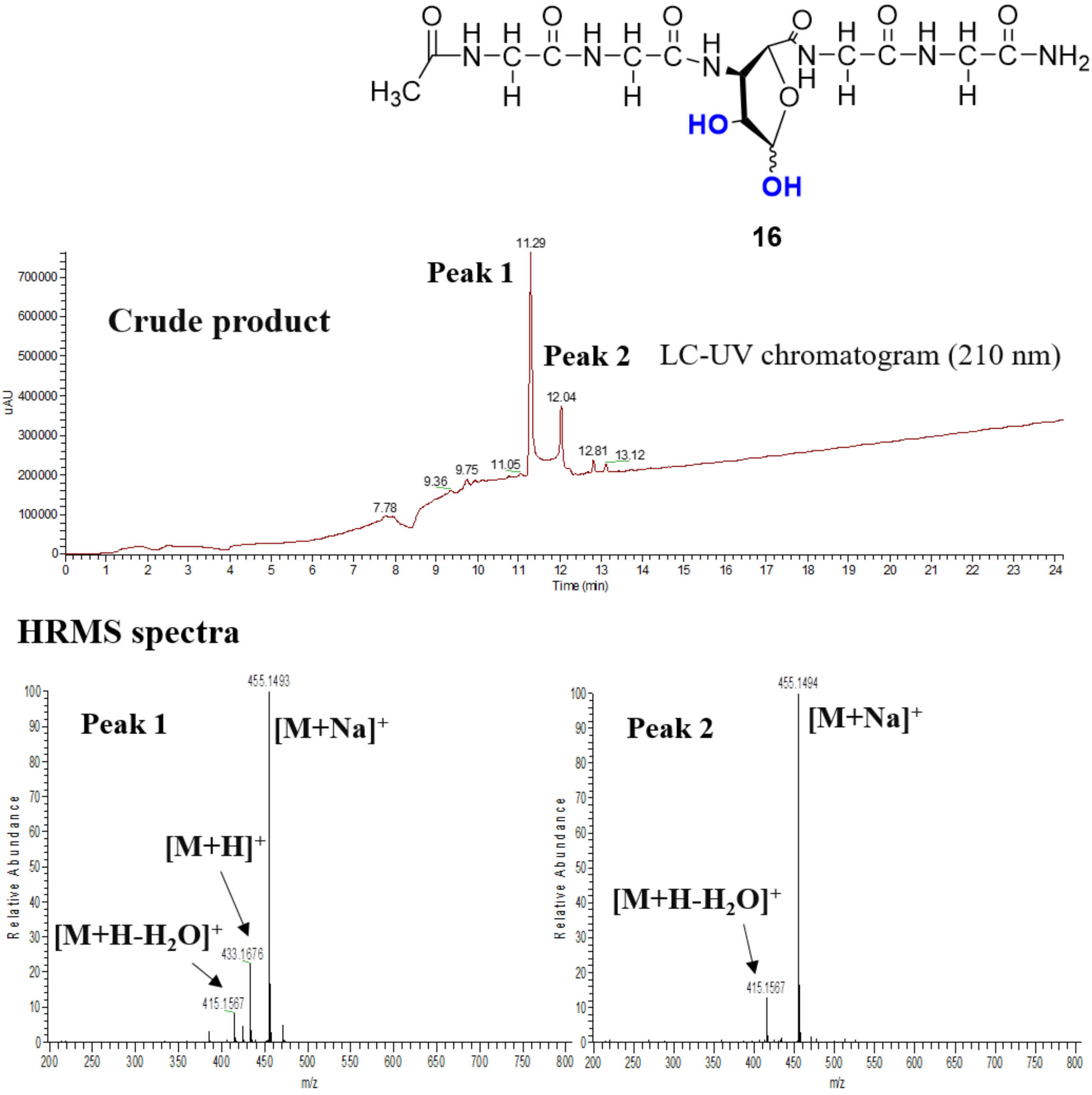

SFig. 16 HILIC LC-UV-MS chromatogram of Ac-Gly-Gly-RibAFU-Gly-Gly-NH_2 (**16**). Retention times are 11.29 min and 12.04 min, presenting a 4:1/α:β anomeric ratio; *m/z* calculated for $C_{15}H_{24}N_6O_9$ $[M+H]^+$ 433.1683 and $[M+Na]^+$ 455.1502, found 433.1676 and 455.1493, respectively

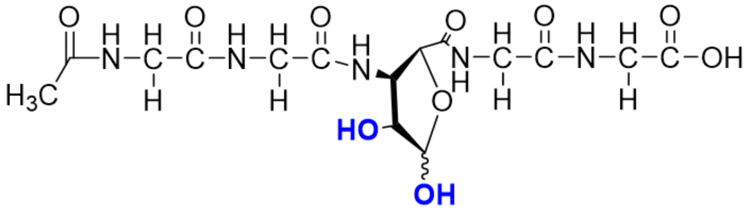

17

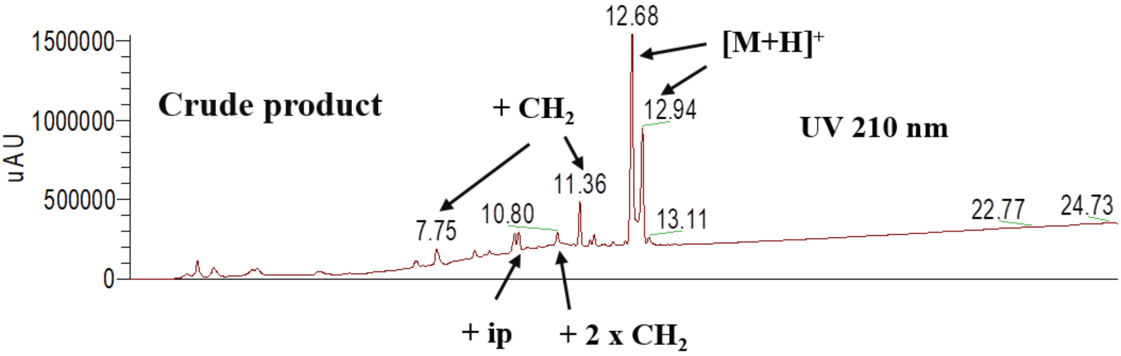

HRMS spectrum

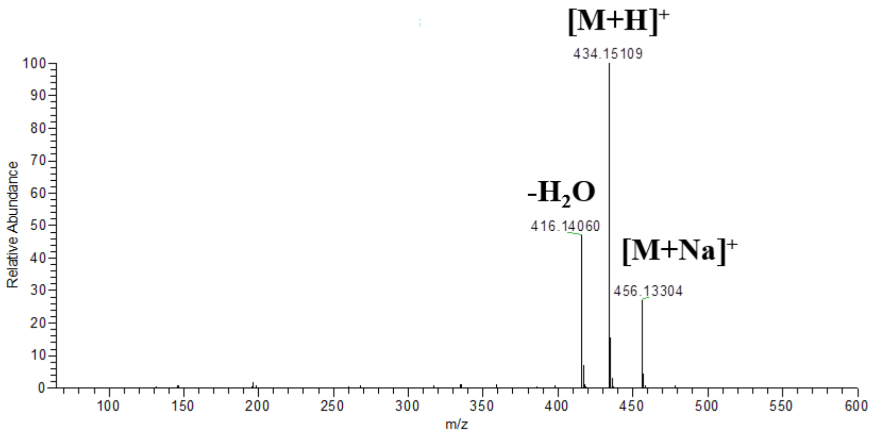

SFig. 17 HILIC LC-UV-MS chromatogram of Ac-Gly-Gly-RibAFU-Gly-Gly-OH (**17**). Retention times are 12.68 min and 12.94 min, presenting a 2:1/α:β anomeric ratio; *m/z* calculated for $C_{15}H_{23}N_5O_{10}$ $[M+H]^+$ 434.15232, $[M+Na]^+$ 456.13426 and $[M+H-H_2O]^+$ 416.14176, found 434.15109, 456.413304 and 416.14060, respectively

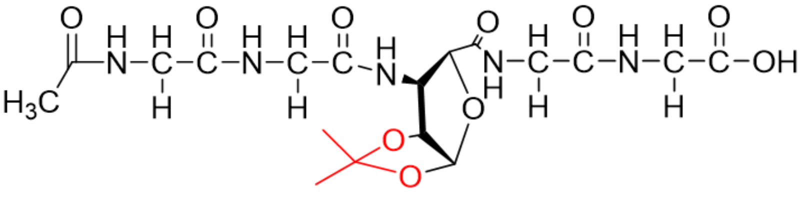

18

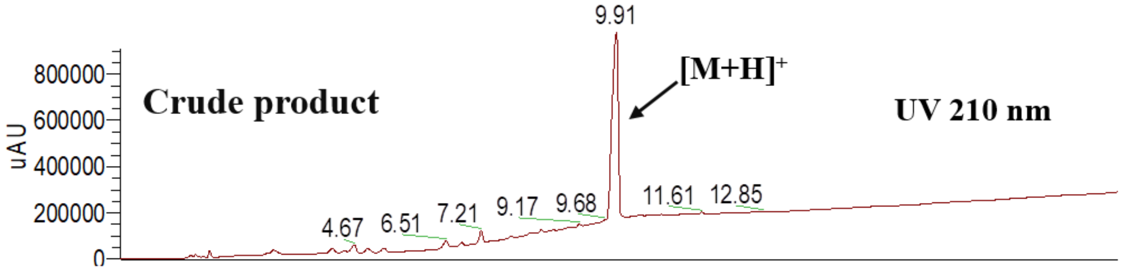

HRMS spectrum

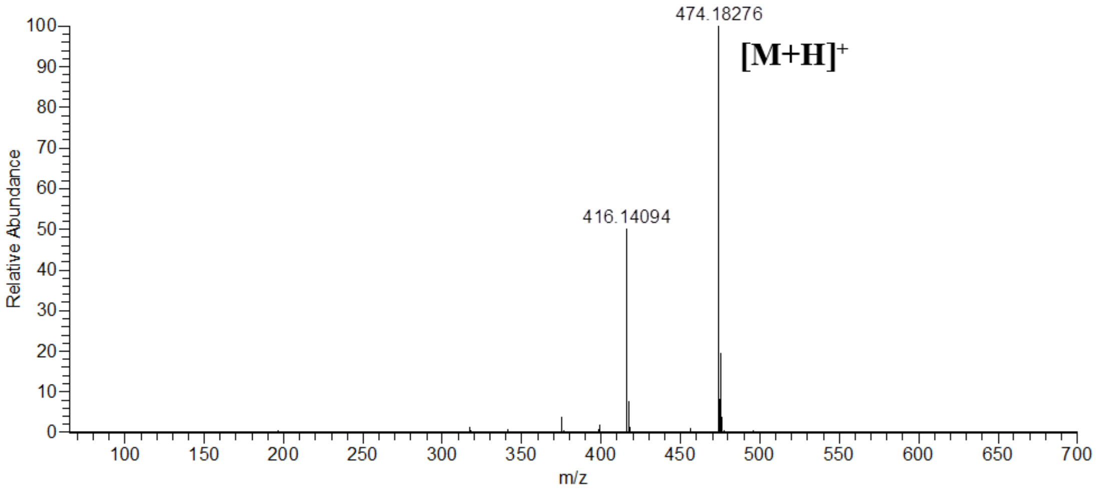

SFig. 18 HILIC LC-UV-MS chromatogram of Ac-Gly-Gly-RibAFU(ip)-Gly-Gly-OH (**18**). Retention time: 9.91 min; *m/z* calculated for $C_{18}H_{27}N_5O_{10}$ $[M+H]^+$ 474.18362, found 474.18276

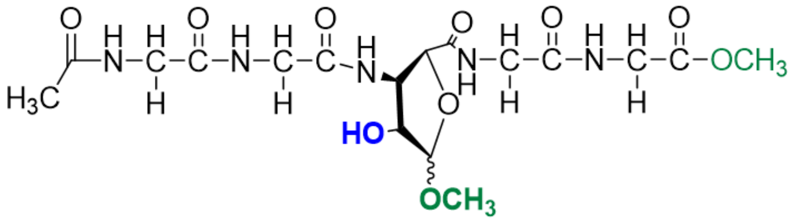

19

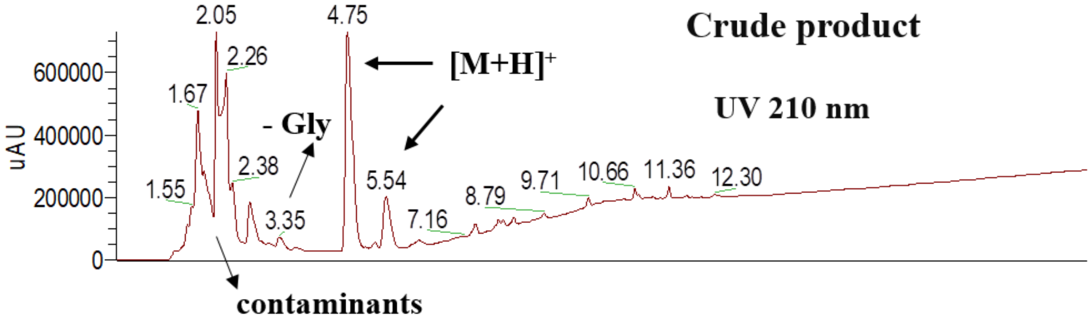

HRMS spectrum

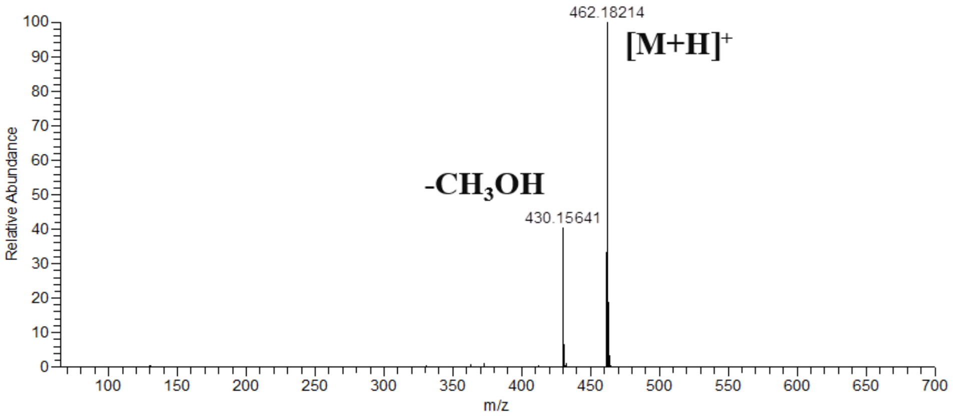

SFig. 19 HILIC LC-UV-MS chromatogram of Ac-Gly-Gly-RibAFU(Me)-Gly-Gly-OMe (**19**) in condition of IR- 120 H^+ (8 eqv.)/MeOH, 60 ºC, 3 h with starting **17**. Retention times are 4.75 min and 5.54 min, presenting a 4:1/α:β anomeric ratio; *m/z* calculated for $C_{17}H_{27}N_5O_{10}$ $[M+H]^+$ 462.18362 and $[M+H-CH_3OH]^+$ 430.15741, found 462.18214 and 430.15641, respectively

RP-HPLC chromatograms for following deprotection

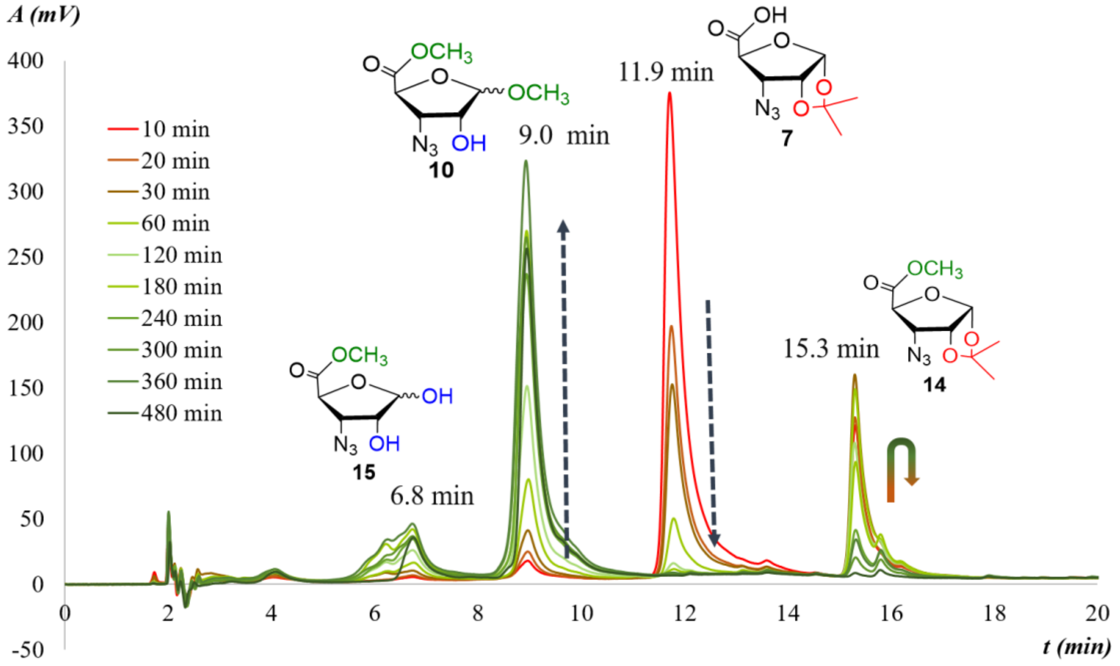

SFig. 20 The 1,2-*O*-isopropylidene removal from the N_3-RibAFU(ip)-OH as function of the time resolved by RP-HPLC: (**7**) treated with 4 eqv. of Amberlite IR-120 H^+ resin in MeOH at 60 ºC

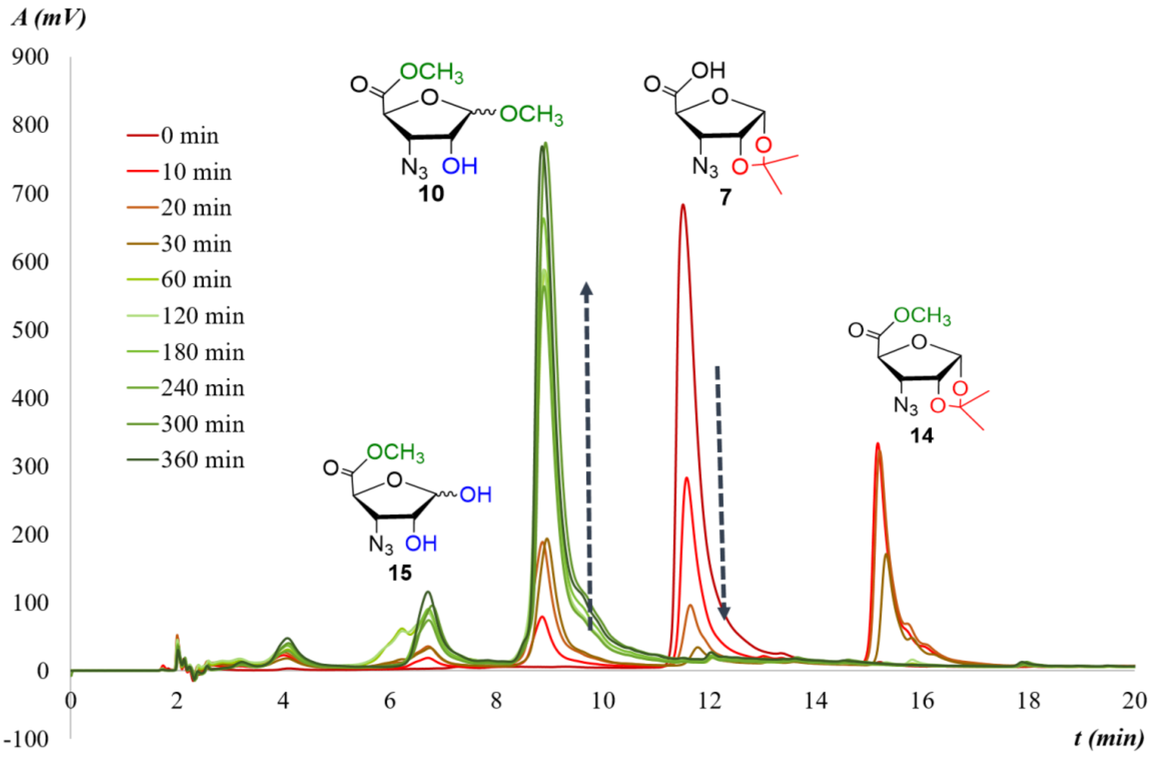

SFig. 21 The 1,2-*O*-isopropylidene removal from the N_3-RibAFU(ip)-OH as function of the time resolved by RP-HPLC: (**7**) treated with 12 eqv. of Amberlite IR-120 H^+ resin in MeOH at 60 ºC

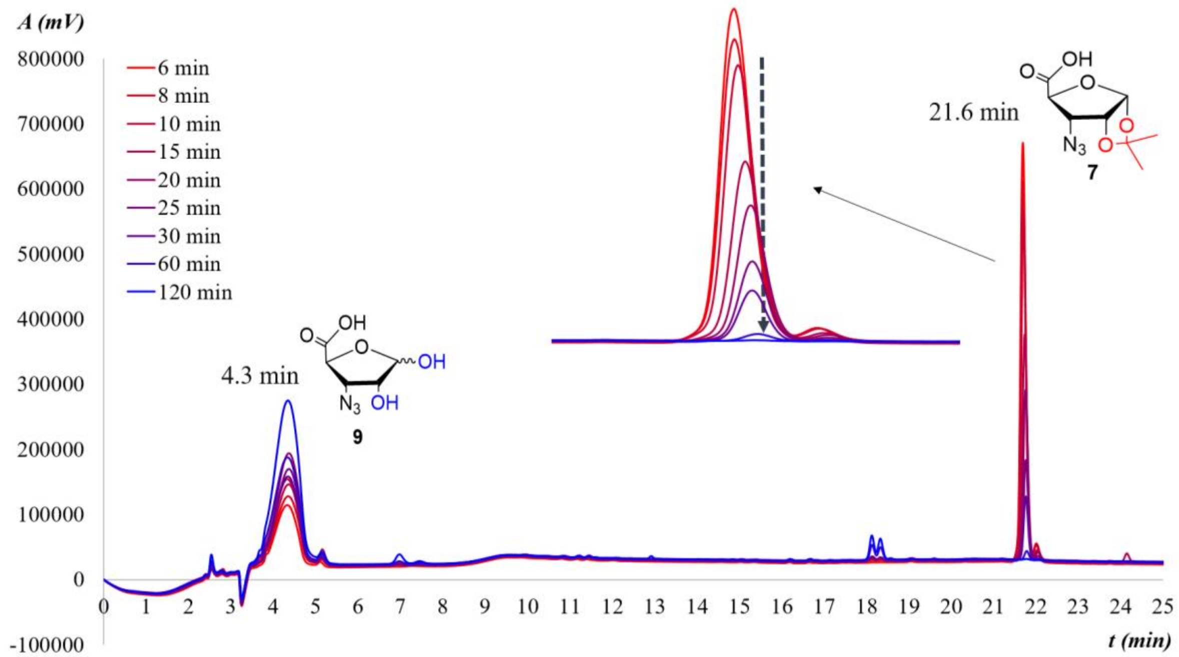

SFig. 22 The 1,2-*O*-isopropylidene removal from the N_3-RibAFU(ip)-OH as function of the time resolved by RP-HPLC: (**7**) treated with 50% TFA in DCM/TIS/H_2O

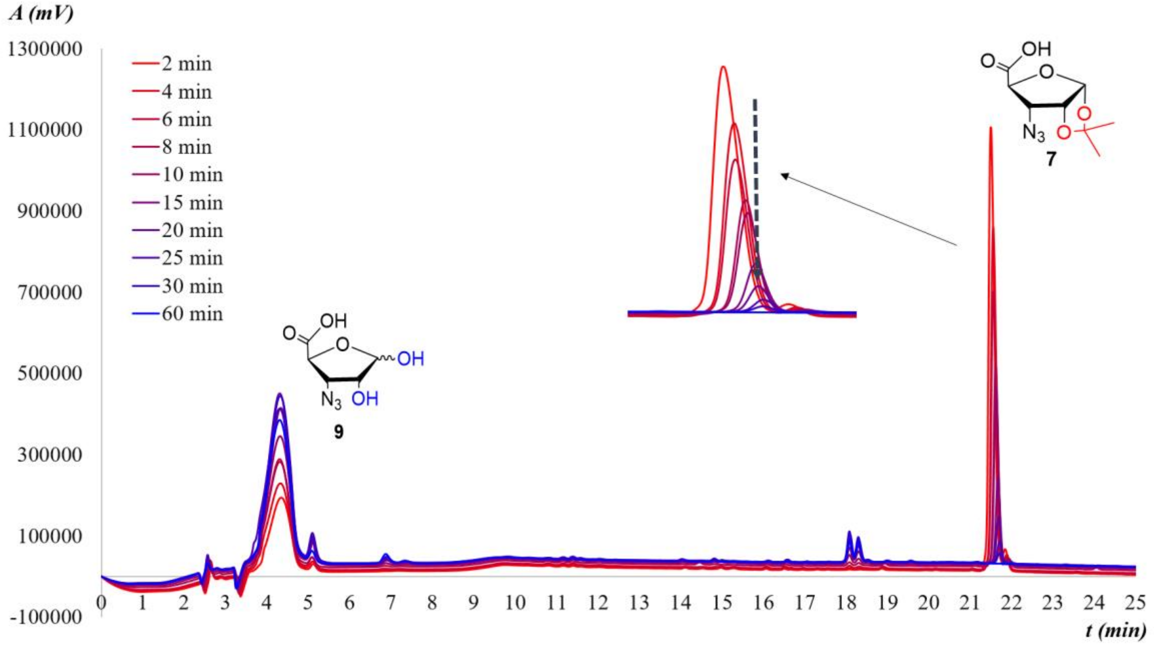

SFig. 23 The 1,2-*O*-isopropylidene removal from the N_3-RibAFU(ip)-OH as function of the time resolved by RP-HPLC: (**7**) treated with 70% TFA in DCM/TIS/H_2O

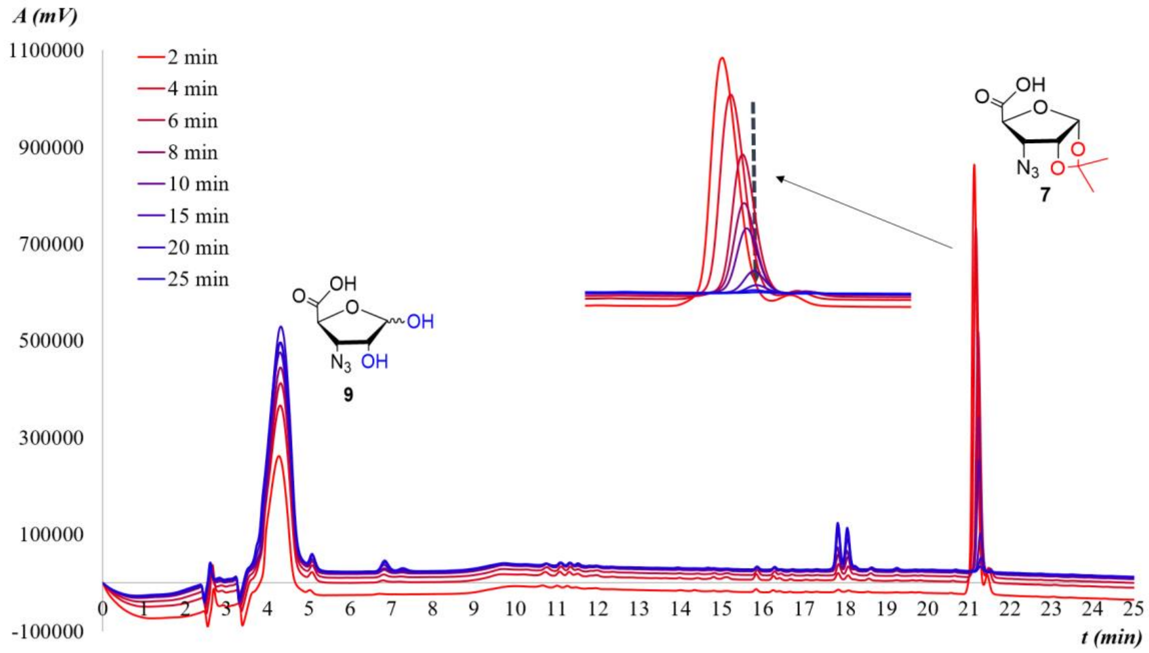

SFig. 24 The 1,2-*O*-isopropylidene removal from the N_3-RibAFU(ip)-OH as function of the time resolved by RP-HPLC: (**7**) treated with 90% TFA in DCM/TIS/H_2O

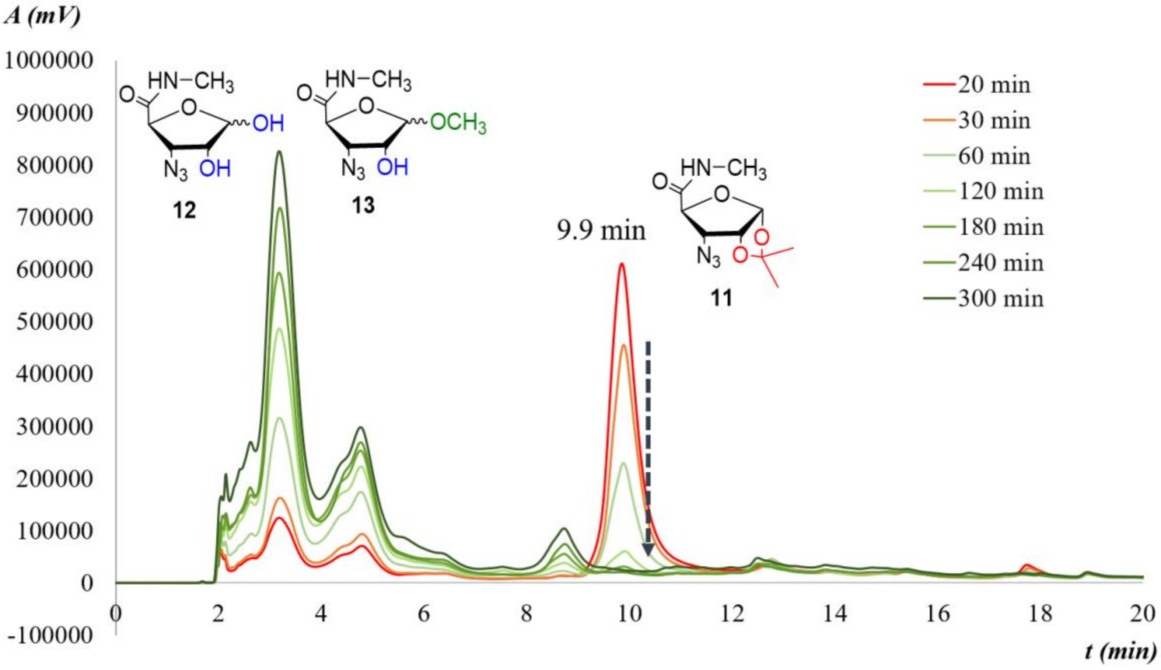

SFig. 25 The 1,2-*O*-isopropylidene removal from the N_3-RibAFU(ip)-NHMe as function of the time resolved by RP-HPLC: (**11**) treated with 8 eqv. of Amberlite IR-120 H^+ resin in MeOH at 60 ^oC

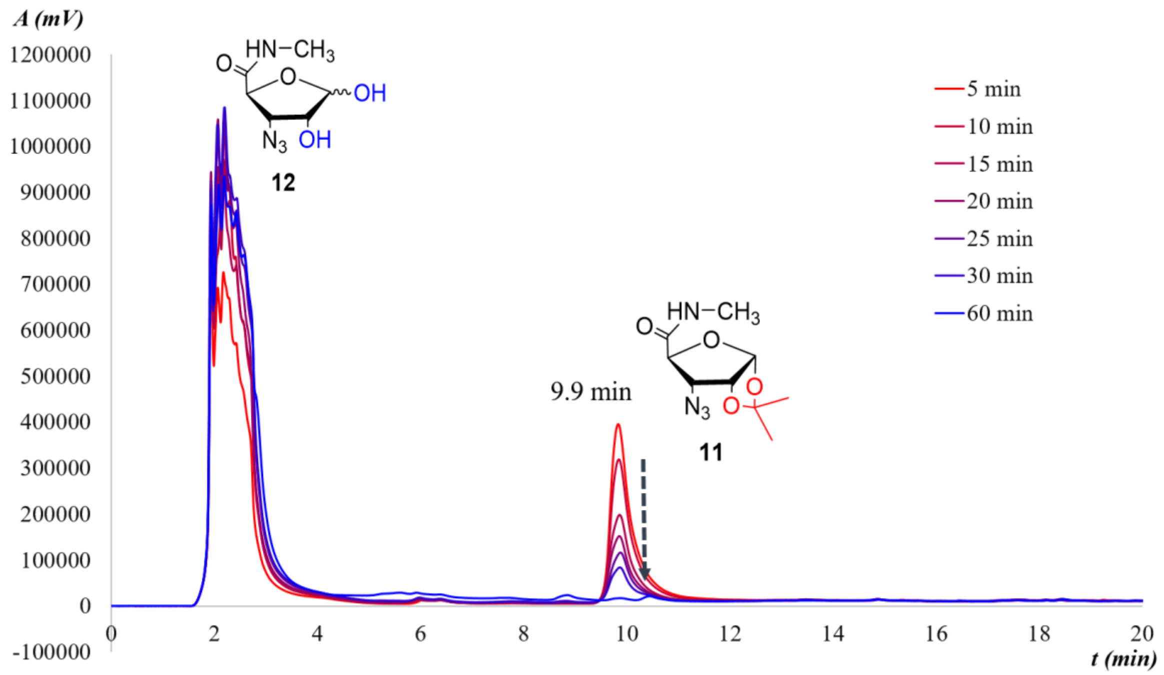

SFig. 26 The 1,2-*O*-isopropylidene removal from the N_3-RibAFU(ip)-NHMe as function of the time resolved by RP-HPLC: (**11**) treated with 50% TFA in DCM/TIS/H_2O

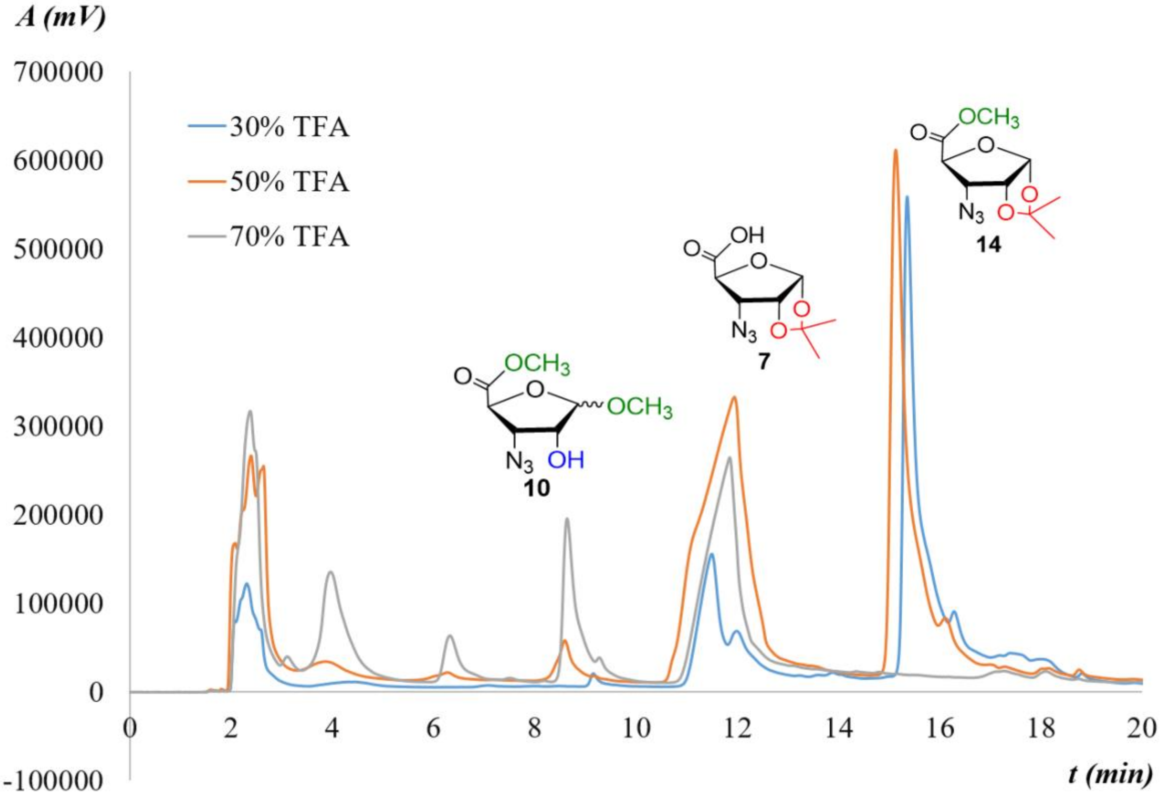

SFig. 27 The 1,2-*O*-isopropylidene removal from the N_3-RibAFU(ip)-OH as function of the time resolved by RP-HPLC: (**7**) treated with different concentration of TFA in MeOH for 18 h

Mechanism for deprotection components

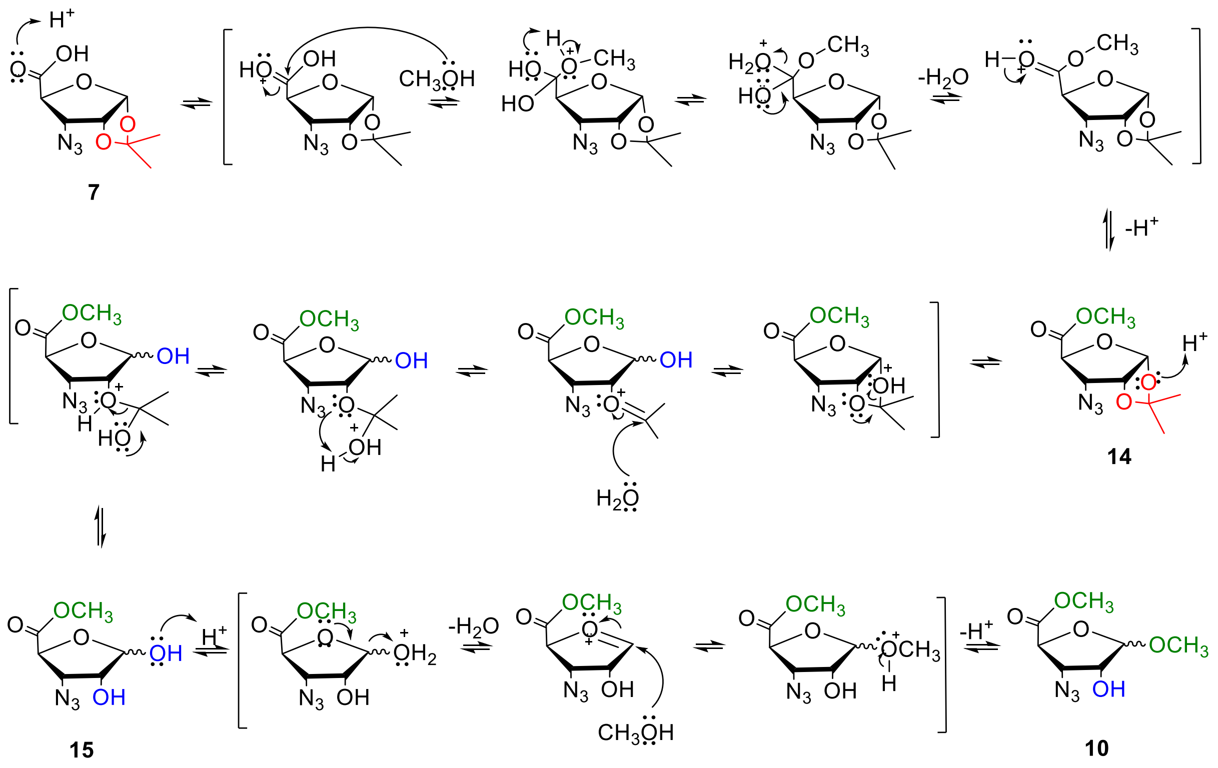

SScheme 1 Proposed mechanism on the removal of 1,2-*O*-isopropylidene from model SAA (**7**). The parallel changing of starting **7**, product **10**, and intermediates (**14**, **15**) in deprotection was determined in different retention times on the RP-HPLC results. The compounds **7** and **10** were identified by ^{1}H NMR and ESI-MS

Tables

STable 1 Changes of the concentrations in deprotection mixture with Amberlite IR-120 H^+ (8 eqv.) in MeOH at 40 ºC

	**Components in reaction mixture (%)**			
**Time (min)**	Starting azido derivative **7**	Intermediate **14**	Intermediate **15**	Main product **10**
10	88	12	n.d.	n.d.
20	76	24	n.d.	n.d.
30	64	36	n.d.	n.d.
60	37	57	n.d.	6
120	13	68	n.d.	19
180	4	58	5	33
240	2	49	6	43
300	2	25	8	65
360	<1	34	8	58
480	n.d.[a]	26	8	66
1440 (1 day)	n.d.	n.d.	10	90
[a] Components become too low to detect them by RP-HPLC chromatography				

STable 2 Changes of the concentrations in deprotection mixture with Amberlite IR-120 H^+ (8 eqv.) in MeOH at RT

	**Components in reaction mixture (%)**			
**Time (min)**	Starting azido derivative **7**	Intermediate **14**	Intermediate **15**	Main product **10**
10	94	6	n.d.	n.d.
20	89	11	n.d.	n.d.
30	83	17	n.d.	n.d.
60	67	33	n.d.	n.d.
120	41	57	n.d.	2.0
180	25	71	n.d.	4
240	16	77	n.d.	7
300	10	80	n.d.	10
360	7	80	n.d.	13
480	3.0	76.5	n.d.	20.5
1440 (1 day)	n.d.[a]	37.5	5.9	56.6
2880 (2 day)	n.d.	12.9	8.5	78.6
5760 (4 day)	n.d.	2.5	9.2	88.3
[a] Components become too low to detect them by RP-HPLC chromatography				
